# Network dynamics of depression, anxiety, sleep disturbances, and suicidal symptoms in Chinese adolescents: a longitudinal cross-sectional and cross-lagged panel network analysis

**DOI:** 10.1017/S0033291726103183

**Published:** 2026-01-23

**Authors:** Bin Sun, Jie Zhang, Yarong Ma, Hongbo He

**Affiliations:** 1 The Affiliated Brain Hospital, Guangzhou Medical University, China; 2 Guangdong Engineering Technology Research Center for Translational Medicine of Mental Disorders, China; 3 Shenzhen Second People’s Hospital, China; 4 Guangdong Provincial People’s Hospital Affiliated to Southern Medical University, China

**Keywords:** adolescent mental health, cross-lagged panel network, depression networks, sleep disturbances, suicidal ideation

## Abstract

**Background:**

Depression in adolescents involves complex interactions among depression, anxiety, sleep disturbances, and suicidal symptoms. Network theory offers insights into dynamic symptom relationships during recovery.

**Methods:**

Of 797 adolescents initially enrolled, 649 with complete baseline data were included in the network analyses; 458 and 277 participants were retained at the 1-month and 3-month follow-ups, respectively. Cross-sectional Gaussian Graphical Models and Cross-Lagged Panel Network (CLPN) analyses examined relationships among nine symptom domains: depression, somatic/subjective anxiety, sleep quantity/quality, daytime insomnia, passive/active sleepiness, and suicidal ideation/tendency. Network centrality and bootstrap validation assessed parameter stability.

**Results:**

Cross-sectional networks showed structural invariance across timepoints (p>0.05). Subjective anxiety demonstrated highest centrality at T0-T1, while somatic symptoms dominated at T2. Depression maintained high closeness centrality throughout. Although betweenness centrality also suggested a central role for depression, its lower stability (CS < 0.5) necessitates a more cautious interpretation of this specific metric. CLPN revealed more predictive relationships during T0→T1 (76.5% significant edges) than T1→T2 (24.7%). Active sleepiness strongly predicted subsequent somatic anxiety (B=0.683) and depression (B=0.647). Suicide ideation-tendency showed stable bidirectional connections. Network stability was excellent (CS>0.5) except betweenness centrality.

**Conclusions:**

Central symptoms evolved during recovery, with subjective anxiety initially dominant but somatic symptoms becoming central over time. The early post-treatment period showed heightened symptom network activity, with sleep disturbances identified as robust predictors of subsequent affective deterioration. Findings support dynamic, network-informed interventions targeting evolving symptom centrality and predictive pathways, particularly addressing sleep-related symptoms and suicide risk during critical recovery phases.

## Introduction

Major depressive disorder represents a leading cause of disability worldwide among adolescents, associated with profound personal suffering, impaired functioning, and elevated suicide risk (Collaborators, 2022; Hauenstein, 2003). Critically, depression in youth rarely presents in isolation. It manifests as a complex syndrome characterized by high comorbidity with anxiety symptoms, pervasive sleep disturbances (including insomnia and hypersomnolence), and clinically significant suicidal ideation or behaviors (Kessler, Avenevoli, & Ries Merikangas, 2001; Lopez-Castroman & Jaussent, 2020; Solmi et al., 2022). This intricate constellation of symptoms contributes to a heterogenous and often persistent clinical presentation, posing substantial challenges for effective intervention and relapse prevention (Birmaher et al., 2007). Traditional approaches, focusing on latent common factors or investigating associations between isolated symptoms and global severity, offer limited insight into how specific symptoms (e.g. somatic anxiety, subjective worry, daytime fatigue from insomnia, passive vs. active sleepiness, suicidal thoughts vs. preparatory acts) dynamically interact within the individual to maintain and potentially exacerbate the disorder over time (Borsboom & Cramer, 2013; Fried & Nesse, 2015).

The network theory of psychopathology provides a powerful alternative conceptual framework (Borsboom, 2017; Cramer, Waldorp, van der Maas, & Borsboom, 2010). This theory posits that mental disorders emerge from a causal web of interactions among symptoms. Within this system, symptoms are conceptualized as nodes within a network, and their pairwise causal or functional relationships constitute the edges connecting them. The structure of this network determines the dynamics of the disorder. Centrality metrics identify symptoms acting as highly influential ‘hubs’ (e.g. High Expected Influence or Strength) or critical ‘bridges’ (e.g. High Betweenness) connecting different symptom clusters. These central symptoms are theorized to play pivotal roles in maintaining the overall network and may represent prime targets for therapeutic intervention (S. Epskamp, Borsboom, & Fried, 2018; Robinaugh, Millner, & McNally, 2016). Empirically, cross-sectional symptom network analyses using methods like Gaussian Graphical Models (GGM) estimated with graphical LASSO have revealed unique patterns of symptom interplay in adult depression (Beard et al., 2016; Fried et al., 2014) and, increasingly, in adolescent samples (Banos-Chaparro, 2024; Liu et al., 2024a,b). These studies highlight core symptoms and influential connections, such as the centrality of mood and sleep items. However, static networks capture associations at a single point and cannot elucidate the temporal dynamics or directional influences between symptoms.

Understanding how clusters of depressive, anxious, sleep-related, and suicidal symptoms influence each other across developmentally sensitive periods requires longitudinal data and analytical methods capable of inferring directionality. Cross-Lagged Panel Network (CLPN) models address this gap (Epskamp et al., 2018; Wysocki et al., 2025). By combining regularized regression (e.g. LASSO) with panel data, CLPNs estimate potential directional paths – specifically, autoregressive effects (a symptom predicting itself later) and cross-lagged effects (one symptom predicting a different symptom later) – while controlling for concurrent associations and covariates. Metrics like in-expected influence (in-EI) and out-expected influence (out-EI) further quantify the relative susceptibility or influence of each node within the directed network over time. Despite the potential of CLPNs, their application to adolescent depression remains scarce (Funkhouser et al., 2021; Zhang, Huang, & Xu, 2024), particularly in tracking the co-evolution of multifaceted depressive, anxious, sleep (e.g. insomnia dimensions, hypersomnolence subtypes), and suicidal (ideation vs. tendency) symptoms across multiple critical post-treatment intervals. Furthermore, the stability and robustness of identified networks in these populations require rigorous assessment.

To address these gaps, this study employed an integrated symptom network approach in a clinical sample of adolescents with depression. We aimed to map the structure and temporal dynamics linking core symptom domains – depression, anxiety (somatic and subjective facets), sleep disturbances (insomnia-related quantity/quality, daytime impairment, and passive/active hypersomnolence), and suicidal dimensions (ideation and tendency) – at three pivotal time points: baseline hospital admission (T0), and 1-month (T1) and 3-month (T2) post-treatment follow-ups. Specifically, we addressed the following research questions: (1) Structure: What is the core structure (central symptoms and strongest connections) of the cross-sectional symptom networks at T0, T1, and T2? Are these global network structure and overall connectivity strength invariant across time points? (2) Dynamics: What are the directional predictive relationships between these symptoms across the intervals T0→T1 and T1→T2? Which symptoms exert the strongest outgoing influence on others, and which are most susceptible to incoming influence within the directed longitudinal networks? (3) Robustness: How stable and accurate are the estimated parameters in both the cross-sectional and cross-lagged panel networks?

By integrating these network methodologies with bootstrap validation within a longitudinal prospective clinical cohort, this research aims to elucidate symptom interactions in adolescent depression during the critical post-treatment phase. Findings may help identify potential maintaining factors and inform the selection of intervention targets based on their network influence, contributing to strategies for disrupting maladaptive symptom pathways and potentially improving long-term outcomes.

## Methods

### Participants

The data for this study were derived from a prospective multicenter cohort study conducted in Guangzhou, China. This research aimed to evaluate short-term (30-day) and medium-term (90-day) symptom changes and influencing factors among young patients with depression following hospital-based treatment. Participants aged 15–24 years were diagnosed with depressive episode, recurrent depressive disorder, or bipolar disorder with current depressive episode according to ICD-10 criteria. Patients were recruited from inpatient and outpatient departments of the Guangzhou Medical University Affiliated Brain Hospital and Guangdong Provincial People’s Hospital between January 1, 2022, and December 31, 2023. Exclusion criteria included: (1) inability to understand or complete study questionnaires; (2) physical, cognitive, or intellectual disabilities preventing participation; or (3) failure to complete baseline assessments. All enrolled patients underwent evaluation of depressive, anxiety, sleep, and suicide risk symptoms at baseline admission (T0), one month post-treatment (T1), and three months post-treatment (T2). Written informed consent was obtained from all participants after detailed explanation of research procedures. This study was approved by the Institutional Review Board (IRB) of Guangzhou Medical University Affiliated Brain Hospital.

### Symptom assessment

This study evaluated symptoms including depression, somatic anxiety, subjective anxiety, sleep quantity and quality, daytime insomnia symptoms (i.e. daytime impairment caused by insomnia), passive sleepiness and active sleepiness, suicidal ideation, and suicide tendency. The assessment tools were as follows: The Beck Depression Inventory (13-item, BDI-13) (Beck, Rial, & Rickels, 1974) total score evaluated depression severity; the Beck Anxiety Inventory (21-item, BAI-21) (Beck, Epstein, Brown, & Steer, 1988) was assessed through its two dimensions—somatic symptoms and subjective anxiety symptoms; the Athens Insomnia Scale (AIS) (Soldatos, Dikeos, & Paparrigopoulos, 2000) evaluated sleep quantity/quality and daytime insomnia symptoms (representing insomnia-induced daytime symptoms); the Epworth Sleepiness Scale (ESS) (Pilcher et al., 2018) measured passive sleepiness (sleepiness tendency in low-demand situations) and active sleepiness (sleepiness tendency in high-demand situations); and the Beck Scale for Suicide Ideation (BSI) (Xian-Yun et al., 2011) assessed suicidal ideation and suicide tendency dimensions.

The use of composite symptom domains was motivated by methodological and conceptual factors. Methodologically, aggregating items from the self-report scales into a smaller number of domains was necessary to avoid overfitting and to estimate a stable network model, given the sample size. This practice helps ensure the reliability of the identified symptom interactions. Conceptually, the selected domains correspond to distinct, clinically meaningful constructs, which aids in the interpretation of the network structure.

### Data analysis

To explore both the cross-sectional and predictive associations over time among depression, anxiety, sleep disturbances, and suicidal ideation, a series of cross-sectional network and cross-lagged panel network (CLPN) analyses was employed. As a preliminary step, all symptom-domain totals were mean-centred within each wave prior to any network estimation. Then, we independently constructed cross-sectional symptom networks using Gaussian graphical models (GGM) at each assessment wave (T0, T1, T2). The GGM for each wave, comprising nine nodes (representing symptoms), was estimated using the Extended Bayesian Information Criterion graphical LASSO (EBICglasso) method, which models the conditional dependence (partial correlation) relationships among symptoms at that specific time point. We characterized the structure of these symptom networks by computing standardized node centrality measures: Expected Influence (EI) quantifies a symptom’s potential aggregate impact by summing the edge weights connected to it (considering sign); Strength represents the overall intensity of direct connections by summing the absolute values of its edge weights; Closeness reflects the average shortest path distance, inversely indicating how easily the symptom can influence or be influenced by others; Betweenness measures how frequently a symptom lies on the shortest paths connecting other symptom pairs, identifying pivotal ‘bridge’ symptoms. To investigate potential structural differences in the symptom networks across the three assessments, we applied the Network Invariance Test (NIT) and the Global Strength Invariance Test (GSIT), with the significance level adjusted using the Bonferroni method. The formulas for calculating Expected Influence, Strength, Closeness, and Betweenness are as follows:

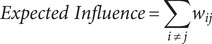




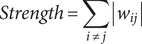




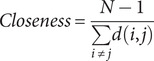




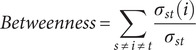

Here, *w_ij_* represents the weight of the edge between the node *i* and the node *j*, *N* is the total number of nodes, *d(i,j)* is the shortest path distance between nodes *i* and *j* (defined as the sum of the reciprocals of the absolute values of the edge weights along the path), *σ_st_* is the total number of shortest paths between nodes *s* and *t*, and *σ_st_(i)* is the number of these shortest paths that pass through node *i.*

For the primary longitudinal analysis, we implemented two-stage CLPN modeling (T0→T1 and T1→T2) to analyze predictive associations. The CLPN models were constructed as follows: For each symptom Y at time t, a multivariate LASSO regularized linear regression model was fitted, predicting Y(t) from all nine symptoms at time t-1. This estimates autoregressive effects (the effect of symptom X at t-1 on itself at t) and cross-lagged effects (the effect of symptom X at t-1 on a different symptom Y at t), while controlling for all other symptoms at t-1 and covariates (age, gender, education level, disease duration). For each node-specific regression, tenfold cross-validation with the deviance statistic was used to select the optimal LASSO penalty parameter (lambda, λ), choosing the largest λ value falling within one standard error of the minimum (λ.1se) to prioritize model parsimony and robust predictors. The estimated coefficients (autoregressive on the matrix diagonal, cross-lagged off-diagonal) from these regressions formed the weighted adjacency matrices for each lag interval. To characterize directional influence within these networks, we computed node-level in-expected influence (in-EI; sum of incoming edge weights, indicating susceptibility to prior influence) and out-expected influence (out-EI; sum of outgoing edge weights, quantifying potential aggregate outgoing influence on subsequent symptoms). For analytical consistency and comparability, both in-EI and out-EI values were standardized using z-scores within each assessment wave prior to interpretation. The specific calculation formulas for in-Expected Influence and out-Expected Influence are as follows:

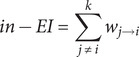




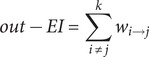

Here, *k* is the number of other nodes, *w*_*i*→*j*_ is the cross-lagged coefficient representing node *i* predicting node *j*, and *w*_*j*→*i*_ is the cross-lagged coefficient representing node *j* predicting node *i.*

To evaluate the accuracy and stability of the estimated parameters in both the cross-sectional and CLPN networks, we implemented nonparametric bootstrap validation procedures based on 1,000 random bootstrap samples. For both network types, 95% confidence intervals were computed for all edge weights. For cross-sectional networks specifically, significance tests for differences in node centrality indices and specific edge weights between assessment waves were performed. Additionally, the stability of centrality indices in cross-sectional networks was assessed through case-dropping bootstrap subset analyses, reported as the correlation stability (CS) coefficient. The CS coefficient quantifies the maximum proportion of cases that can be dropped while maintaining at least a 0.70 correlation between centrality indices computed from the full and subsetted datasets with 95% probability. Typically, CS coefficients > 0.50 indicate excellent stability, coefficients between 0.25 and 0.50 indicate moderately acceptable stability, and coefficients < 0.25 suggest the stability estimate is insufficient for firm conclusions.

To evaluate the potential impact of loss to follow-up on the study results, we conducted a sensitivity analysis using multiple imputation. This study generated 20 imputed datasets and pooled them, subsequently repeating all primary analyses (including network estimation, centrality measures, and edge weight calculation) using the pooled data. By computing Spearman rank correlation coefficients between the imputed data and the original data for centrality indices, edge weights, and cross-lagged paths, and comparing the consistency of the identified core symptoms and key pathways, we systematically assessed the robustness of the findings to missing data.

All analyses were conducted using R software (version 4.4.3). Cross-sectional network estimation, centrality quantification, and all bootstrap stability procedures (edge CIs, CS coefficients) were performed using the *bootnet* package (Epskamp et al., 2018). Comparisons of network structure across time (NIT, GSIT) were conducted with the *NetworkComparisonTest* package (van Borkulo et al., 2023). The core LASSO regression models for the CLPN analysis were estimated using the *glmnet* package (Friedman, Hastie, & Tibshirani, 2010). Network visualizations for both cross-sectional and CLPN analyses were generated using the *qgraph* package (Epskamp et al., 2012). For the multiple imputation sensitivity analysis, the *mice* package (van Buuren & Groothuis-Oudshoorn, 2011) was utilized.

## Results

### Descriptive statistics

Supplementary Table S1 presents the symptoms and their labels used in the network analysis of this study, along with their corresponding assessment tools and dimensions. A total of 797 patients were enrolled in the study. Descriptive data at each time point were derived from the subset of participants with complete symptom assessments: 649 at baseline (T0), 458 at the one-month follow-up (T1), and 277 at the three-month follow-up (T2). Table 1 displays the demographic characteristics, medicine treatment, and symptoms of these samples at the three time points, along with the average levels of the nine symptoms. The attrition analysis revealed evolving patterns of baseline differences between retained and lost participants over time (Supplementary Table S2). At the T0 assessment, participants lost to follow-up had a shorter illness duration (1.9 ± 2.0 vs. 2.5 ± 2.2 years, p=0.003) and a lower rate of benzodiazepine use (39.2% vs. 43.5%, p=0.038). At the T1 follow-up, those lost were younger (18.1 ± 3.3 vs. 18.7 ± 3.1 years, p=0.004), had a different distribution of education levels (p=0.001) with a lower average education level, and showed lower use of mood stabilizers (28.6% vs. 36.9%, p=0.014) and antipsychotics (45.7% vs. 54.1%, p=0.019). At T2, participants lost to follow-up were younger (18.2 ± 3.3 vs 18.8 ± 3.1 years, p = 0.012) and lower average education level (p = 0.001) than those who remained. No significant differences were found for gender at any time point.

**Table 1.** Demographic characteristics and symptoms of each subsample

Variables	T0 (N=649)	T1 (N=458)	T2 (N=277)
Age	18.4 (3.3)	18.7 (3.1)	18.8 (3.1)
Gender			
Male	150 (23.1%)	101 (22.1%)	60 (21.7%)
Female	499 (76.9%)	357 (77.9%)	217 (78.4%)
Education			
Junior High or Lower	154 (23.7%)	97 (21.3%)	51 (18.5%)
High School	246 (37.9%)	153 (33.6%)	92 (33.3%)
University or Above	249 (38.4%)	206 (45.2%)	133 (48.2%)
Duration	2.5 (2.2)	2.4 (2.2)	2.3 (2.1)
Antidepressants	422 (65%)	303 (66.2%)	191 (69%)
Benzodiazepines	282 (43.5%)	199 (43.4%)	117 (42.2%)
Mood Stabilizers	215 (33.1%)	169 (36.9%)	107 (38.6%)
Antipsychotics	331 (51%)	248 (54.1%)	153 (55.2%)
Depression symptom	20.2 (8.9)	17.7 (10.4)	15.6 (10.4)
Somatic symptom	12.3 (7.9)	10.5 (7.9)	8.8 (7.6)
Subjective anxiety	12.5 (6.7)	10.6 (6.9)	9.2 (6.8)
Sleep quantity and quality	6.6 (3.9)	5.2 (3.5)	4.8 (3.3)
Daytime insomnia symptoms	4.5 (2.2)	4 (2.3)	3.6 (2.1)
Passive Sleepiness	8.4 (4.6)	8.4 (4.6)	7.9 (4.7)
Active Sleepiness	1.4 (1.6)	1.4 (1.5)	1.1 (1.4)
Suicide Ideation	4.5 (3.3)	4 (3.2)	3.4 (3.1)
Suicide tendency	7.1 (8)	6.1 (7.6)	4.9 (7.5)

*Note:* T0 = baseline. T1 = one-month follow-up. T2 = three-month follow-up.

Regarding baseline symptom comparisons between completers and those lost to follow-up, results differed between timepoints. At T1, no significant differences were found in any of the nine symptoms. At T2, significant differences emerged in eight symptoms except daytime insomnia symptoms. Completers showed lower baseline scores than non-completers for depression (18.8±8.8 vs 20.8±8.9, p=0.01), somatic (11.2±7.9 vs 12.8±7.8, p=0.018), anxiety (11.6±6.7 vs 13.0±6.6, p=0.013), sleep quality (6.2±3.8 vs 6.9±4.0, p=0.047), passive sleepiness (7.8±4.6 vs 8.7±4.5, p=0.019), active sleepiness (1.1±1.4 vs 1.5±1.6, p=0.003), suicidal ideation (4.0±3.3 vs 4.8±3.3, p=0.009) and suicidal tendency (5.9±7.7 vs 7.7±8.1, p=0.006).

### Cross-sectional networks

**Figure 1.**
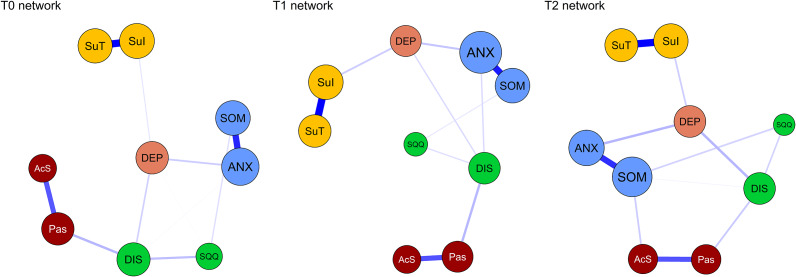
The cross-sectional networks for T0 (left), T1 (middle) and T2 (right). Blue edges represent positive associations, with thickness indicating relationship strength. Node sizes are proportional to strength centrality, highlighting highly connected symptoms. For clarity, only edges with an absolute weight > 0.1 are shown. *Note:* DEP, Depression symptoms; SOM, Somatic symptom; ANX, Subjective anxiety; SQQ, Sleep quantity and quality; DIS, Daytime insomnia symptoms; Pas, Passive Sleepiness; AcS, Active Sleepiness; SuI, Suicide Ideation; SuT, Suicide tendency.

Figure 1 displays cross-sectional symptom networks at baseline (T0), 1-month follow-up (T1), and 3-month follow-up (T2). Both the Global Strength Invariance test and Network Structure Invariance test demonstrated statistically invariant symptom networks across the three waves (all p-values > 0.05). Comprehensive results of these invariance analyses are documented in Supplementary Table S3.

**Figure 2.**
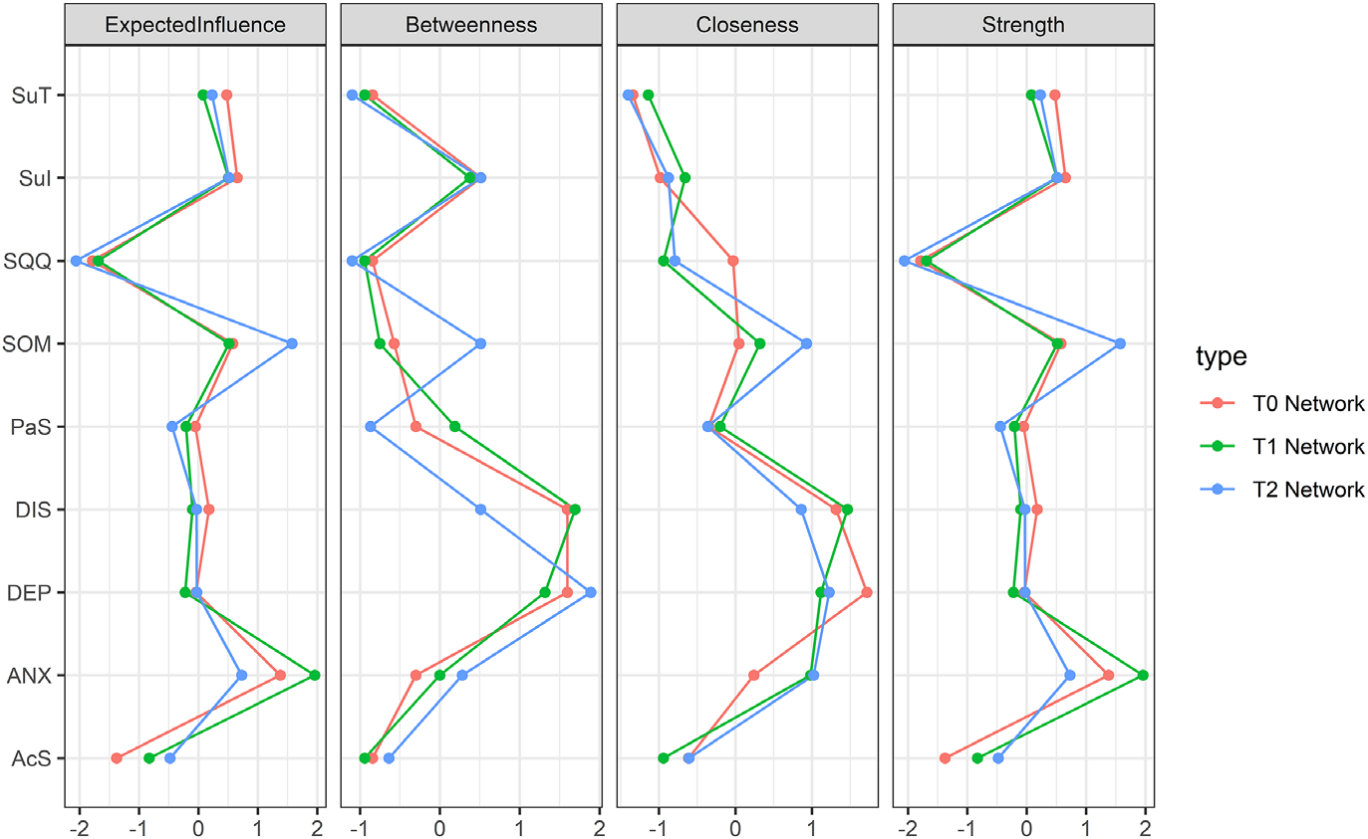
Standardized centrality indices based on cross-sectional networks from T0 to T2. *Note:* DEP, Depression symptoms; SOM, Somatic symptom; ANX, Subjective anxiety; SQQ, Sleep quantity and quality; DIS, Daytime insomnia symptoms; Pas, Passive Sleepiness; AcS, Active Sleepiness; SuI, Suicide Ideation; SuT, Suicide tendency.

Figure 2 presents standardized centrality estimates for the cross-sectional symptom networks. During the first two waves, centrality estimates exhibited comparable patterns: ‘Subjective Anxiety’ demonstrated the highest Expected Influence (EI) and Strength, while ‘Depression’ and ‘Daytime Insomnia Symptoms’ ranked highest in Closeness and Betweenness. However, at the third wave (T2), ‘Somatic Symptoms’ surpassed ‘Subjective Anxiety’ to become the dominant symptom in EI and Strength, whereas ‘Depression’ remained the most central in Closeness and Betweenness. Supplementary Figure S2 displays the statistical significance of inter-group differences in these centrality metrics.

At baseline, 19 edges (52.8%) showed positive and non-zero correlations; at T1, 16 edges (44.4%) were positive and non-zero correlated; at T2, 13 edges (36.1%) exhibited positive non-zero correlations. Supplementary Figure S1 reveals that the strongest positive correlations across all three waves consistently occurred between ‘Suicide Ideation’ and ‘Suicide Tendency’, ‘Somatic Symptoms’ and ‘Subjective Anxiety’, as well as ‘Passive Sleepiness’ and ‘Active Sleepiness’.

Supplementary Figure S3 presents the stability analysis results of network parameters across three cross-sectional waves. The CS coefficients for all networks demonstrated values exceeding 0.5 in edge (CS-E-T0=0.75, CS-E-T1=0.751, CS-E-T2=0.751), expected influence (CS-EI-T0=0.672, CS-EI-T1=0.751, CS-EI-T2=0.596), strength (CS-S-T0=0.672, CS-S-T1=0.672, CS-S-T2=0.516), and closeness (CS-C-T0=0.672, CS-C-T1=0.751, CS-C-T2=0.516). However, the betweenness centrality exhibited lower stability with CS coefficients ranging between 0.25–0.5 across time points (CS-B-T0=0.439, CS-B-T1=0.36, CS-B-T2=0.282). These results indicate that the connective characteristics of core nodes—including edge density, expected influence, strength, and closeness—remained stable across all three time points, whereas the stability of betweenness centrality progressively declined yet remained within acceptable levels.

### Cross-lagged panel networks

**Figure 3.**
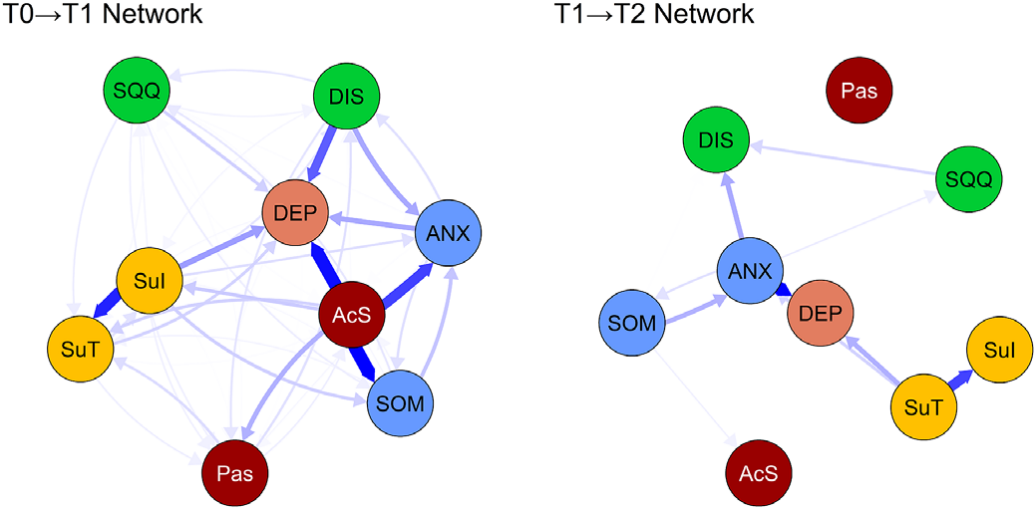
The cross-lagged panel networks for T0→T1 (left), and T1→T2 (right). Arrows represent unique longitudinal relationships. The blue colored lines indicate positive associations between the nodes, and thicker lines indicate stronger relationships between the nodes. *Note:* DEP, Depression symptoms, SOM, Somatic symptom, ANX, Subjective anxiety, SQQ, Sleep quantity and quality, DIS, Daytime insomnia symptoms, Pas, Passive Sleepiness, AcS, Active Sleepiness, SuI, Suicide Ideation, SuT, Suicide tendency.

Figure 3 displays the cross-lagged panel symptom networks for transitions from T0 to T1 and T1 to T2, adjusted for age, gender, education level, and disease duration, while excluding autoregressive effects to enhance visual interpretability of the cross-lagged paths. Cross-lagged and autoregressive edge weights are detailed in Supplementary Tables S4, S5 and Supplementary Figure S4. In the T0→T1 network, 62 edge weights (76.5%) showed significant positive non-zero values, with an average non-zero weight of B=0.123. In contrast, in the T1→T2 network, only 20 edge weights (24.7%) exhibited positive non-zero values, averaging B=0.046.

Supplementary Figure S5 displays the edge weight accuracy in the cross-lagged networks for T0→T1 and T1→T2, while Supplementary Figure S6 shows bootstrap difference tests for edge weights across these networks. The three strongest cross-lagged paths in the T0→T1 network were: Active Sleepiness (AcS)→Somatic Symptoms (SOM; B=0.683), AcS→Depression Symptoms (DEP; B=0.647), and Suicide Ideation (SuI)→Suicide Tendency (SuT; B=0.590). In contrast, only Suicide Tendency (SuT)→Suicide Ideation (SuI; B=0.155) was identified as the strongest path in the T1→T2 network.

**Figure 4.**
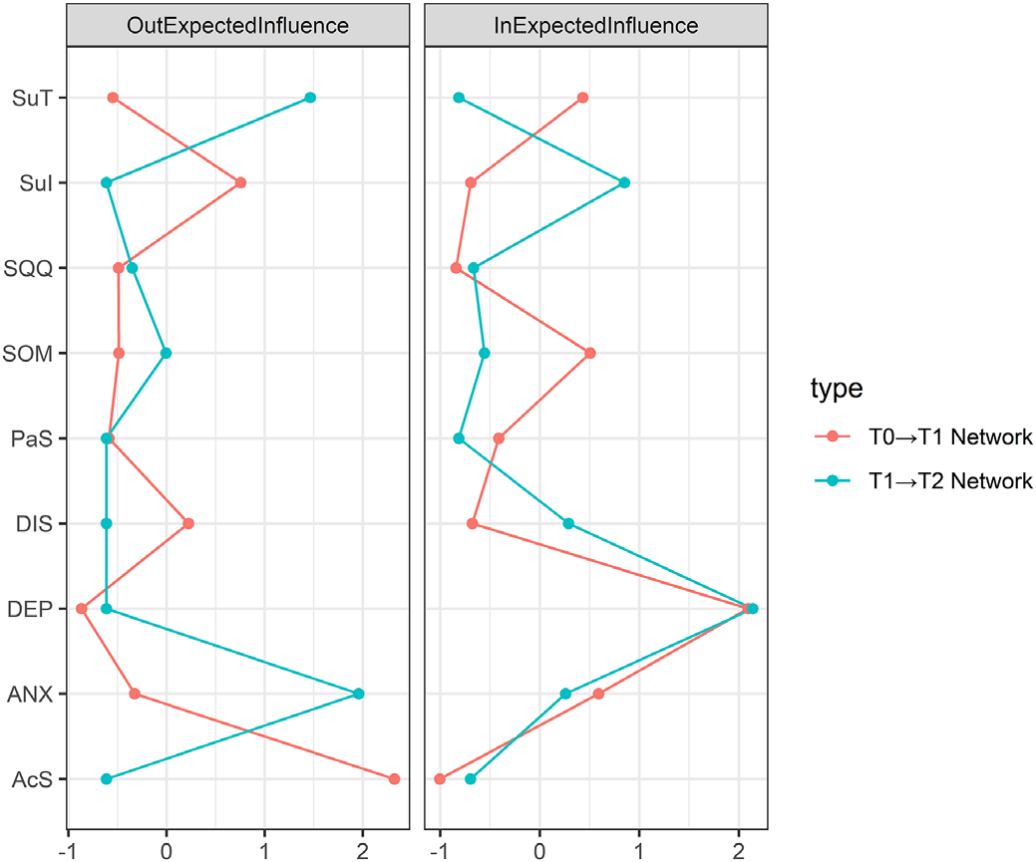
Symptom centrality estimates in the T0→T1 and T1→T2 network. Larger values reflect greater centrality. *Note:* DEP, Depression symptoms, SOM, Somatic symptom, ANX, Subjective anxiety, SQQ, Sleep quantity and quality, DIS, Daytime insomnia symptoms, Pas, Passive Sleepiness, AcS, Active Sleepiness, SuI, Suicide Ideation, SuT, Suicide tendency.

Figure 4 presents the standardized centrality estimates derived from the cross-lagged panel networks. Supplementary Figure S7 displays bootstrapped difference tests for these centrality indices across the two CLPNs. In both CLPNs, ‘Depression symptoms’ (DEP) consistently exhibited the highest in-expected influence (in-EI). The strongest in-EI symptoms beyond DEP included ‘Subjective Anxiety’ (ANX), ‘Somatic symptoms’ (SOM), and ‘Suicide Tendency’ (SuT) in the T0→T1 network, and ‘Suicide Ideation’ (SuI) and ‘Daytime Insomnia symptoms’ (DIS) in the T1→T2 network. Regarding the strongest out-expected influence (out-EI), ‘Active Sleepiness’ (AcS) and ‘Suicide Ideation’ (SuI) were dominant in T0→T1, whereas ‘Subjective Anxiety’ (ANX) and ‘Suicide Tendency’ (SuT) were strongest in T1→T2.

Supplementary Figure S8 presents the stability analysis of network parameters for both CLPNs, revealing substantial differences. Stability was higher in the T0→T1 network: in-EI showed good stability (CS-inEI = 0.749), while edge weights (CS-E = 0.362) and out-EI (CS-outEI = 0.284) exhibited acceptable stability. In contrast, all stability coefficients in the T1→T2 network fell below 0.25 (CS-inEI = 0.128; CS-E = 0.204; CS-outEI = 0.204), indicating that interpretations of centrality indices and edge rankings in this network require caution.

### Sensitivity analysis

To evaluate the potential impact of missing data on our findings, we conducted a sensitivity analysis using multiple imputation. The original datasets at T1 and T2 were re-analyzed following multiple imputation, and the resulting network structures (including centrality indices and edge weights) were compared to the original complete-case analyses to assess robustness.

The sensitivity analysis for the cross-sectional network estimates revealed high consistency between the original and pooled data. For centrality metrics, Pearson correlations were strong at both T1 (Strength = 0.933, Betweenness = 0.719, Closeness = 0.950, Expected Influence = 0.933) and T2 (Strength = 0.967, Betweenness = 0.923, Closeness = 0.967, Expected Influence = 0.967) (Supplementary Figure S9). The rankings of the most central symptoms remained virtually unchanged (Supplementary Table S6). Similarly, the edge weights were highly correlated across networks (T1: r =0 .979; T2: r =0.933) (Supplementary Figure S10), with the strongest symptom-symptom connections being identical. These results confirm that the cross-sectional network structures are robust to missing data.

For the cross-lagged panel network (CLPN) analyses, the sensitivity to missing data varied between the two time intervals. The T0→T1 CLPN demonstrated high robustness, with strong correlations between original and imputed data for both in-expected and out-expected influence centrality (r = 0.95 and r = 0.90, respectively). The rankings of the most central symptoms remained largely consistent post-imputation. In contrast, the T1→T2 CLPN, particularly for in-expected influence (r = 0.544), showed greater variability, with more substantial shifts in the centrality rankings of key symptoms such as SuI and SOM (Supplementary Figure S11, Supplementary Table S7). Similarly, the cross-lagged paths were highly stable in the T0→T1 network (r = 0.923), whereas the T1→T2 network exhibited lower consistency (r = 0.612), although the strongest path (SuT→SuI) was consistently identified in both original and imputed data (Supplementary Figure S12). These findings indicate that the cross-lagged relationships from T0→T1 are highly robust to missing data, while the results from T1→T2 should be interpreted with greater caution.

## Discussion

This study represents the first systematic application of cross-sectional symptom networks and cross-lagged panel network analyses to delineate the complex dynamic relationships between depression, anxiety, sleep disturbances, and suicidal symptoms in Chinese adolescents with depression across baseline, one-month, and three-month post-treatment intervals. This longitudinal network perspective not only reveals the instantaneous correlational structure among symptoms but also clarifies their temporal sequences of mutual prediction and influence.

First, we found that across all assessment waves, depression, anxiety, sleep disturbances, and suicidal symptoms remained highly interconnected, with the overall network structure and connection strength demonstrating remarkable long-term stability. Critically, our multiple imputation sensitivity analyses confirmed that these cross-sectional network structures were robust to missing data, with strong correlations between original and imputed centrality measures (r > 0.90 at T1/T2) and virtually unchanged symptom rankings. These findings align with recent network studies in both adult (Beard et al., 2016; Fried et al., 2014) and adolescent (Banos-Chaparro, 2024; Liu et al., 2024a,b) depression samples, further substantiating the applicability of network theory to psychiatric disorders (Borsboom, 2017; Cramer et al., 2010). The structural invariance tests confirmed that the global network architecture remained consistent across time points, suggesting that the fundamental symptom interplay patterns persist despite potential changes in overall severity levels during recovery.

More specifically, an evolution in network centrality was observed, primarily based on the most stable indices (Expected Influence and Strength). Subjective anxiety demonstrated the highest centrality at baseline and one-month follow-up, but by the three-month assessment, somatic symptoms became dominant. This shift was supported by robust stability coefficients for EI and Strength (CS > 0.5). Meanwhile, depression consistently exhibited high closeness centrality. The betweenness centrality of depression, while suggesting a potential bridging role, showed limited stability over time (CS < 0.5), and thus findings related to this index should be interpreted as preliminary. This temporal shift in network centrality suggests that the ‘core’ symptoms within adolescent depression networks may evolve with recovery progression, indicating that intervention targets should be dynamically adjusted according to network dynamics. The persistent centrality of depression symptoms as a network hub underscores its fundamental role in maintaining the overall symptom system, consistent with theoretical conceptualizations of depression as a self-reinforcing network of symptoms (Bringmann et al., 2013; Fried, 2017).

Regarding direct symptom associations, the strongest positive correlations consistently occurred between suicide ideation and suicide tendency, somatic symptoms and subjective anxiety, and passive sleepiness and active sleepiness across all waves. These findings not only corroborate clinical observations but also align with the pathological coupling within symptom spectra (Kessler et al., 2001; Lopez-Castroman & Jaussent, 2020). The robust connection between different facets of suicidality highlights the importance of comprehensive suicide risk assessment in adolescent depression, while the strong anxiety symptom interconnections suggest shared underlying mechanisms that may warrant integrated treatment approaches (Cummings, Caporino, & Kendall, 2014; Garber & Weersing, 2010).

The granular subdivision of sleep disturbances into quantity/quality, daytime insomnia, passive sleepiness, and active sleepiness was empirically grounded and enhanced our ability to capture these associations. Sleep quantity/quality and daytime insomnia represent core components of insomnia disorders per ICD-10 criteria (Soldatos et al., 2000), while the passive/active sleepiness distinction derives from factor analyses of the Epworth Sleepiness Scale (ESS) in depressive populations (Lundt, 2005; Pilcher et al., 2018). This approach allowed us to differentiate between functional dimensions of sleep problems: for instance, passive sleepiness (e.g. in low-demand situations) may reflect hypersomnolence linked to amotivation in depression (Demyttenaere, De Fruyt, & Stahl, 2005), whereas active sleepiness (e.g. in high-demand situations) could indicate attentional deficits (Lim & Dinges, 2010).

In the CLPN analyses, the T0→T1 interval revealed more extensive and stronger predictive relationships compared to T1→T2, indicating that the first month post-discharge represents a sensitive period of heightened symptom system activity and reorganization. Our sensitivity analyses further validated these temporal patterns: the T0→T1 network demonstrated high robustness (cross-lagged path stability r=0.923), with active sleepiness maintaining its strong predictive effects on subsequent somatic anxiety and depression symptoms despite missing data imputation. In contrast, the T1→T2 network showed greater variability (cross-lagged path stability r=0.612), though the suicide tendency→ideation pathway remained consistently identified. This specificity aligns with network theory’s emphasis on symptom-level interactions—active sleepiness (e.g. in high-demand situations) may serve as a bridge symptom that precipitates broader affective deterioration through cognitive pathways (e.g. executive dysfunction), underscoring the value of granular sleep subdomains in identifying precise intervention targets. Active sleepiness demonstrated the strongest temporal associations with subsequent somatic anxiety and depression symptoms, while suicide ideation strongly predicted suicide tendency, emphasizing that sleep-driven symptoms may constitute important risk factors for the recurrence and exacerbation of core affective symptoms (Lopez-Castroman & Jaussent, 2020; Pilcher et al., 2018; Soldatos et al., 2000). This finding extends previous research linking sleep disturbances to depression maintenance by demonstrating specific temporal pathways through which hypersomnolence symptoms are strongly associated with broader symptom deterioration (Goldstein, Bridge, & Brent, 2008; Lovato & Gradisar, 2014). During the T1→T2 interval, predictive relationships notably weakened except for suicide tendency predicting suicide ideation, potentially reflecting that most patients achieved partial remission over time, resulting in lower network activation, or that external interventions (follow-up care, special attention) exerted positive effects. The bidirectional relationship between suicide ideation and tendency across different time lags suggests a self-perpetuating cycle that may require targeted interruption strategies (Klonsky, May, & Saffer, 2016; O’Connor & Nock, 2014).

A critical consideration in interpreting our findings is the differential stability of network indices. We therefore prioritize the interpretation of the most stable metrics, Expected Influence and Strength (CS>0.5), which provide the most reliable evidence for our conclusions. In contrast, the betweenness centrality index exhibited lower stability (CS<0.5), making its interpretation less reliable. Similarly, the T1→T2 CLPN indices had low stability coefficients (<0.25). Consequently, we have focused our discussion on the robust findings from stable indices and qualified any statements involving less stable parameters. Our sensitivity analyses corroborated this pattern: while cross-sectional centrality showed excellent stability (CS>0.5), the T1→T2 CLPN indices had lower stability coefficients (<0.25), highlighting the need for caution in interpreting later network dynamics. The differential stability across metrics suggests that while core symptom connections remain robust, the specific pathways through which symptoms influence each other may be more variable, potentially reflecting individual differences in recovery trajectories (Fisher, Medaglia, & Jeronimus, 2018; Fried et al., 2017).

Our findings offer several hypotheses for future clinical research that are informed by the network perspective. First, the evolving centrality of subjective and somatic anxiety suggests that monitoring fluctuations in these symptoms may serve as early warning signs of overall clinical deterioration or relapse. Second, the identified predictive roles of active sleepiness and suicide ideation position them as promising candidate targets for management and monitoring. For instance, one could hypothesize that targeting active sleepiness (e.g. via carefully timed interventions in high-demand situations) might prevent cascading effects on affective symptoms, whereas addressing passive sleepiness (e.g. through behavioral activation) could reduce a motivation. Finally, the dynamic nature of symptom centrality implies that intervention strategies may need to be adapted according to recovery stage. These network-informed hypotheses support the development of personalized treatment approaches that target symptoms based on their evolving influence (Hofmann & Hayes, 2019; Lutz et al., 2018), though their efficacy awaits direct testing in future intervention trials.

It is important to note that while our cross-lagged panel network analysis identifies directional predictive relationships, it does not permit definitive causal inferences. The observed associations, however robust, could be influenced by unmeasured common causes or confounding variables. Therefore, the identified pathways (e.g. from active sleepiness to depression) should be interpreted as strong, temporally-prioritized hypotheses that merit future experimental or interventional validation.

While we have reported the proportion of participants receiving various psychotropic medications at each time point, the present study did not collect detailed information on treatment duration, dosage, or psychotherapeutic interventions prior to or during the follow-up period. Consequently, the observed changes in symptom severity and network structure may reflect treatment effects rather than the natural course of adolescent depression. For example, the prominent role of active sleepiness in predicting subsequent depressive and somatic symptoms at T0→T1 could be influenced by sedating effects of antidepressants or antipsychotics, rather than intrinsic symptom dynamics. Similarly, the apparent shift from subjective to somatic anxiety centrality over time might be attenuated by anxiolytic or antidepressant effects, rather than a true reconfiguration of symptom hierarchy. Therefore, the temporal patterns identified in our cross-lagged panel networks should be interpreted as symptom trajectories under naturalistic treatment conditions, rather than pure disease progression. Future studies should prospectively record treatment type, dose, adherence, and response, and ideally integrate treatment nodes into the network models to disentangle pharmacological effects from symptom-symptom interactions.

Several limitations warrant consideration. First, the relatively high attrition rate at follow-up may have underestimated some long-term symptom associations, potentially biasing results toward individuals with better treatment engagement or outcomes. However, our multiple imputation sensitivity analyses demonstrated that core network structures and key pathways (particularly in T0→T1) remained robust despite missing data, mitigating concerns about selection bias. Second, the generalizability of our findings may be limited by the specific nature of our clinical sample—Chinese adolescents seeking treatment in specialized psychiatric settings. The symptom networks identified may be influenced by culturally specific factors (e.g. academic pressure) and healthcare-system factors (e.g. help-seeking pathways). Third, network analyses were based on self-report questionnaires and scale subscores, unable to encompass multilevel covariates such as family support, social functioning, or biological markers that may moderate symptom relationships (Jones, Ma, & McNally, 2021; McElroy et al., 2018). Fourth, while LASSO regularization enhances model sparsity and robustness, it may underestimate weaker but clinically meaningful pathways in small samples or among highly correlated variables (Epskamp et al., 2018; Wysocki et al., 2025). Additionally, the exclusive focus on symptom-level variables precludes examination of how external factors (e.g. life events, treatment adherence) may influence network dynamics over time (Snippe et al., 2017; Wichers, 2014). At last, this study was designed as a naturalistic observational cohort, and thus reflects real-world treatment heterogeneity rather than a controlled intervention. While this limits causal inference about symptom dynamics, it enhances ecological validity, as the findings are more representative of routine clinical care in Chinese adolescent psychiatry settings.

Future research should: (1) Employing multicenter samples with diverse cultural backgrounds and extended follow-up periods is essential to validate the generalizability of these network dynamics and to determine the universality versus cultural specificity of the identified symptom pathways; (2) Incorporate cognitive, environmental, and biological markers to construct higher-dimensional symptom influence networks that capture the full complexity of adolescent depression (Kendler et al., 1995; Schmaal et al., 2017); (3) Explore the efficacy of network-informed personalized interventions that target ‘core symptoms’ identified through individual network analyses (Boschloo et al., 2015; Kroeze et al., 2017). Additionally, ecological momentary assessment methods could provide finer-grained temporal resolution to capture within-person network dynamics and their relationship to clinical outcomes (Trull & Ebner-Priemer, 2013).

## Conclusion

This study leverages symptom network analysis to provide novel insight into the complex connections and dynamic interactions among multiple symptoms over time in Chinese adolescents with depression. By identifying central symptoms and their temporal predictive patterns, our findings offer evidence for precision medicine approaches to adolescent psychological intervention and risk screening, potentially informing the development of more targeted and effective treatment strategies.

## Supporting information

10.1017/S0033291726103183.sm001 Sun et al. supplementary material 1 Sun et al. supplementary material

10.1017/S0033291726103183.sm002 Sun et al. supplementary material 2 Sun et al. supplementary material

## Supplementary material

The supplementary material for this article can be found at http://doi.org/10.1017/S0033291726103183.
