# Supplementary material for: Network dynamics of depression, anxiety, sleep disturbances, and suicidal symptoms in Chinese adolescents: a longitudinal cross-sectional and cross-lagged panel network analysis
Source: Psychol Med. 2026 Jan 23;56:e30. doi: 10.1017/S0033291726103183 (PMC12885338; doi:10.1017/S0033291726103183)
Supplement: Sun et al. supplementary material 1 — Sun et al. supplementary material [file S0033291726103183sup001.docx]

#0. LOADING RELEVANT PACKAGES AND DATASETS

library(haven)

library(bootnet)

library(qgraph)

library(NetworkComparisonTest)

library(psych)

library(glmnet)

library(dplyr)

library(haven)

library(ggplot2)

library(tidyr)

library(scales)

##CROSS-SECTIONAL NETWORKs DATASETs

self_index <- read_sav("self_index.sav")

self_1st <- read_sav("self_1st.sav")

self_3rd <- read_sav("self_3rd.sav")

##CROSS LAGGED PANEL NETWORKs DATASETs

W1W2_Nmissingsav <- read_sav("W1W2._Nmissingsav.sav")

W2W3_Nmissingsav <- read_sav("W2W3._Nmissingsav.sav")

nodelist<- c("DEP", "SOM", "ANX", "SQQ", "DIS", "PaS", "AcS", "SuI", "SuT")

#1.BUILD CROSS-SECTIONAL NETWORKS FROM T0 TO T2.

W1NET<-estimateNetwork(self_index[,c(3:11)],default = "EBICglasso",threshold=TRUE,labels = nodelist)

W2NET<-estimateNetwork(self_1st[,c(2:10)],default = "EBICglasso",threshold=TRUE,labels = nodelist)

W3NET<-estimateNetwork(self_3rd[,c(2:10)],default = "EBICglasso",threshold=TRUE,labels = nodelist)

#1.1 EXPORT NETWORK STRUCTURES TO TIFF FIGURE 1.

tiff("combined_networks.tiff", width=10, height=5, units="in", res=1200, compression="lzw")

layout(t(1:3))

W1NET_plot<-qgraph(W1NET$graph,posCol="blue",negCol="none",fade = TRUE,layout="spring", vsize = 15,

legend=FALSE, groups=list("BDI SCORE"=c(1),"BAI symptoms"=c(2:3),"AIS symptoms"=c(4:5),

"ESS symptoms"=c(6:7),"BSI symptoms"=c(8:9)),

color=c("#E27D60","#6699FF","#00CC33","#990000","#FFC000"),

title="T0 network",

title.cex = 1.5,

mar = c(8, 5, 8, 5),

labels=nodelist,

legend.cex=0.25)

W2NET_plot<-qgraph(W2NET$graph,posCol="blue",negCol="none",fade = TRUE,layout="spring",vsize = 15,

legend=FALSE, groups=list("BDI SCORE"=c(1),"BAI symptoms"=c(2:3),"AIS symptoms"=c(4:5),

"ESS symptoms"=c(6:7),"BSI symptoms"=c(8:9)),

color=c("#E27D60","#6699FF","#00CC33","#990000","#FFC000"),

title="T1 network",

title.cex = 1.5,

mar = c(8, 5, 8, 5),

labels=nodelist,

legend.cex=0.25)

W3NET_plot<-qgraph(W3NET$graph,posCol="blue",negCol="none",fade = TRUE,layout="spring",vsize = 15,

legend=FALSE, groups=list("BDI SCORE"=c(1),"BAI symptoms"=c(2:3),"AIS symptoms"=c(4:5),

"ESS symptoms"=c(6:7),"BSI symptoms"=c(8:9)),

color=c("#E27D60","#6699FF","#00CC33","#990000","#FFC000"),

title="T2 network",

title.cex = 1.5,

mar = c(8, 5, 8, 5),

labels=nodelist,

legend.cex=0.25)

dev.off()

#1.2 DRAW NETWORK CENTRALITY INDICES

tiff("FIG2.tiff", width=8, height=5, units="in", res=1200, compression="lzw")

central<-centralityPlot(list( "T0 Network"=W1NET, "T1 Network"=W2NET,"T2 Network"=W3NET), scale = c("z-scores"), labels=nodelist,

include = c("ExpectedInfluence","Betweenness", "Closeness", "Strength"))

dev.off().

#1.3 NETWORK STABILITY ANALYSIS

Boot_W1NET <- bootnet(W1NET, nCores = 8, nBoots = 1000, type = "nonparametric",

statistics = c("edge", "strength", "closeness", "betweenness","expectedInfluence"))

Boot_W1NET_cs <- bootnet(W1NET, nCores = 8, nBoots = 1000, type = "case",

statistics = c("edge", "strength", "closeness", "betweenness","expectedInfluence"))

Boot_W2NET <- bootnet(W2NET, nCores = 8, nBoots = 1000, type = "nonparametric",

statistics = c("edge", "strength", "closeness", "betweenness","expectedInfluence"))

Boot_W2NET_cs <- bootnet(W2NET, nCores = 8, nBoots = 1000, type = "case",

statistics = c("edge", "strength", "closeness", "betweenness","expectedInfluence"))

Boot_W3NET <- bootnet(W3NET, nCores = 8, nBoots = 1000, type = "nonparametric",

statistics = c("edge", "strength", "closeness", "betweenness","expectedInfluence"))

Boot_W3NET_cs <- bootnet(W3NET, nCores = 8, nBoots = 1000, type = "case",

statistics = c("edge", "strength", "closeness", "betweenness","expectedInfluence"))

pdf("【T0】bootnet—T0.pdf",family="GB1",width=4, height=4)

layout(1)

plot(Boot_W1NET , order = "sample")

plot(Boot_W1NET , "ExpectedInfluence", order = "sample")

plot(Boot_W1NET , "Strength", order = "sample")

plot(Boot_W1NET , "Closeness", order = "sample")

plot(Boot_W1NET , "Betweenness", order = "sample")

dev.off()

pdf("【T1】bootnet—T1.pdf",family="GB1",width=4, height=4)

layout(1)

plot(Boot_W2NET , order = "sample")

plot(Boot_W2NET , "ExpectedInfluence", order = "sample")

plot(Boot_W2NET , "Strength", order = "sample")

plot(Boot_W2NET , "Closeness", order = "sample")

plot(Boot_W2NET , "Betweenness", order = "sample")

dev.off()

pdf("【T3】bootnet—T2.pdf",family="GB1",width=4, height=4)

layout(1)

plot(Boot_W3NET , order = "sample")

plot(Boot_W3NET , "ExpectedInfluence", order = "sample")

plot(Boot_W3NET , "Strength", order = "sample")

plot(Boot_W3NET , "Closeness", order = "sample")

plot(Boot_W3NET , "Betweenness", order = "sample")

dev.off()

pdf("【EdgeS】bootnet.pdf",family="GB1",width=4, height=8)

plot(Boot_W1NET , order = "sample")

plot(Boot_W2NET , order = "sample")

plot(Boot_W3NET , order = "sample")

dev.off()

CS_T0<-corStability(Boot_W1NET_cs)

CS_T1<-corStability(Boot_W2NET_cs)

CS_T2<-corStability(Boot_W3NET_cs)

pdf("Correlation stability from T0 to T2.pdf",family="GB1",width=5, height=5)

plot(Boot_W1NET_cs, statistics = c("Betweenness", "Closeness", "Strength","ExpectedInfluence"))

plot(Boot_W2NET_cs, statistics = c("Betweenness", "Closeness", "Strength","ExpectedInfluence"))

plot(Boot_W3NET_cs, statistics = c("Betweenness", "Closeness", "Strength","ExpectedInfluence"))

dev.off()

#1.4 Comparison of network structures at three time points

nodelist<- c("DEP", "SOM", "ANX", "SQQ", "DIS", "PaS", "AcS", "SuI", "SuT")

colnames(self_index)[3:11]<-nodelist

colnames(self_1st)[2:10]<-nodelist

colnames(self_3rd)[2:10]<-nodelist

W1NET<-estimateNetwork(self_index[,c(3:11)],default = "EBICglasso",threshold=TRUE,labels = nodelist)

W2NET<-estimateNetwork(self_1st[,c(2:10)],default = "EBICglasso",threshold=TRUE,labels = nodelist)

W3NET<-estimateNetwork(self_3rd[,c(2:10)],default = "EBICglasso",threshold=TRUE,labels = nodelist)

T0T1_compare<- NCT(W1NET, W2NET, test.edges=TRUE,

test.centrality=TRUE,p.adjust.methods="fdr",edges="all",

centrality=c("strength", "closeness", "betweenness","expectedInfluence"))

T0T2_compare<- NCT(W1NET, W3NET, test.edges=TRUE,

test.centrality=TRUE,p.adjust.methods="fdr",edges="all",

centrality=c("strength", "closeness", "betweenness","expectedInfluence"))

T1T2_compare<- NCT(W2NET, W3NET, test.edges=TRUE,

test.centrality=TRUE,p.adjust.methods="fdr",edges="all",

centrality=c("strength", "closeness", "betweenness","expectedInfluence"))

#2.BUILD CROSS-LAGGED PANEL NETWORKS (CLPNs).

#2.1 CLPN FOR T0→T1

df11 <- as.matrix(W1W2_Nmising[, c(3:20, 21:23,25)])

df11 <- df11 %>% as.matrix() %>% apply(2, as.numeric)

k <- 9

num_covars <- 4

adjMat <- matrix(0, k, k)

CLPN.fun1 <- function(df11) {

covar_cols <- (2*k + 1):(2*k + num_covars) # 新增

for (i in 1:k) {

set.seed(100) #Create a matrix of independent variables containing covariates,

# but when running bootnet, there cannot be set.seed(100)

predictors <- cbind(

as.matrix(df11[, 1:k]),

as.matrix(df11[, covar_cols])

)

lassoreg <- cv.glmnet(

x = predictors,

y = df11[, k + i],

family = "gaussian",

alpha = 1,

standardize = TRUE,

nfolds = 10

)

lambda <- lassoreg$lambda.1se

coeffs <- coef(lassoreg, s = lambda, exact = FALSE)[2:(k + 1)]

adjMat[1:k, i] <- coeffs

}

return(adjMat)

}

Network_CL_adj <- estimateNetwork(df11, fun = CLPN.fun1, labels = nodelist, directed = TRUE)

#2.2 CLPN FOR T1→T2

W2W3_Nmissingsav <- read_sav("W2W3._Nmissingsav.sav")

df22 <- as.matrix(W2W3_Nmissingsav[, c(2:19, 20:22,24)])

df22 <- df22 %>% as.matrix() %>% apply(2, as.numeric)

k <- 9

num_covars <- 4

adjMat <- matrix(0, k, k)

CLPN.fun2 <- function(df22) {

covar_cols <- (2*k + 1):(2*k + num_covars)

for (i in 1:k) {

set.seed(100) #Create a matrix of independent variables containing covariates,

# but when running bootnet, there cannot be set.seed(100)

predictors <- cbind(

as.matrix(df22[, 1:k]),

as.matrix(df22[, covar_cols])

)

lassoreg <- cv.glmnet(

x = predictors,

y = df22[, k + i],

family = "gaussian",

alpha = 1,

standardize = TRUE,

nfolds = 20

)

lambda <- lassoreg$lambda.1se

coeffs <- coef(lassoreg, s = lambda, exact = FALSE)[2:(k + 1)] # 修改

adjMat[1:k, i] <- coeffs

}

return(adjMat)

}

Network_CL_adj2 <- estimateNetwork(df22, fun = CLPN.fun2, labels = nodelist, directed = TRUE)

##2.3 Draw the cross-lag network diagram (without showing the autoregressive effect).

tiff("FIG3.tiff", width=8, height=5, units="in", res=1200, compression="lzw")

layout(t(1:2))

NETCL_adj<-Network_CL_adj$graph

diag(NETCL_adj) <- 0

netplot_adj<-qgraph(NETCL_adj, abels = nodelist,

posCol="blue",negCol="none",fade = TRUE,layout="spring",vsize = 10,

legend=FALSE, groups=list("BDI SCORE"=c(1),"BAI symptoms"=c(2:3),"AIS symptoms"=c(4:5),

"ESS symptoms"=c(6:7),"BSI symptoms"=c(8:9)),

color=c("#E27D60","#6699FF","#00CC33","#990000","#FFC000"),

title="T0→T1 Network",

title.cex = 1.2, # 字体大小（默认为1）

mar = c(8, 5, 8, 5),

labels=nodelist,

legend.cex=0.25

)

NETCL_adj1<-Network_CL_adj2$graph

diag(NETCL_adj1) <- 0

netplot_adj2<-qgraph(NETCL_adj1, labels = nodelist,

posCol="blue",negCol="red",fade = TRUE,layout="spring",vsize = 10,

legend=FALSE, groups=list("BDI SCORE"=c(1),"BAI symptoms"=c(2:3),"AIS symptoms"=c(4:5),

"ESS symptoms"=c(6:7),"BSI symptoms"=c(8:9)),

color=c("#E27D60","#6699FF","#00CC33","#990000","#FFC000"),

title="T1→T2 Network",

title.cex = 1.2, # 字体大小（默认为1）

mar = c(8, 5, 8, 5),

labels=nodelist,

legend.cex=0.25

)

dev.off()

#2.4 DRAW CLPN CENTRALITY INDICES

tiff("FIG4.tiff", width=6, height=5, units="in", res=1200, compression="lzw")

central_CPLN<-centralityPlot(list( "T0→T1 Network"=Network_CL_adj, "T1→T2 Network"=Network_CL_adj2 ),

labels = nodelist, include = c("OutExpectedInfluence", "InExpectedInfluence"),

scale = c("z-scores"))

dev.off()

#2.5 Analysis of the Stability and Accuracy of CLPN Network,Before running bootnet, the set.seed(100) in the function must be deleted.

Boot_CL_adj <- bootnet(Network_CL_adj, directed = T, nCores = 1, nBoots = 1000,

statistics = c("edge", "outExpectedInfluence", "inExpectedInfluence"))

Boot_CL_CS_adj <- bootnet(Network_CL_adj, nCores = 1, nBoots = 1000, type = "case",

statistics = c("edge", "outExpectedInfluence", "inExpectedInfluence"), directed = T)

Boot_CL_adj2 <- bootnet(Network_CL_adj2, directed = T, nCores = 1, nBoots = 1000,

statistics = c("edge", "outExpectedInfluence", "inExpectedInfluence"))

Boot_CL_CS_adj2 <- bootnet(Network_CL_adj2, nCores = 1, nBoots = 1000, type = "case",

statistics = c("edge", "outExpectedInfluence", "inExpectedInfluence"),

directed = T)

corStability(Boot_CL_CS_adj)

corStability(Boot_CL_CS_adj2)

pdf("CLPNbootnet CS.pdf",family="GB1",width=6, height=6)

plot(Boot_CL_CS_adj, statistics = c( "outExpectedInfluence", "inExpectedInfluence"))

plot(Boot_CL_CS_adj2, statistics = c("outExpectedInfluence", "inExpectedInfluence"))

dev.off()

pdf("CLPNbootnet_edgeline CS.pdf",family="GB1",width=5, height=8)

plot(Boot_CL_adj, order = "sample")

plot(Boot_CL_adj2, order = "sample")

dev.off()

pdf("【T0→T1】CLPNbootnet.pdf",family="GB1",width=6, height=6)

plot(Boot_CL_adj, "edge", plot = "difference", order = "sample")

plot(Boot_CL_adj, "outExpectedInfluence", order = "sample")

plot(Boot_CL_adj, "inExpectedInfluence", order = "sample")

dev.off()

#2.6 Export Cross-lagged LASSO regression predictive effects of T0→T1 and T1→T2 (Table S3 and S4)

Edges_CLPN_t0t1<-Network_CL_adj$graph

Edges_CLPN_t0t1<-as.data.frame(Edges_CLPN_t0t1)

write_xlsx(

x = Edges_CLPN_t0t1,

path = "Edges_CLPN_t0t1.xlsx",

col_names = TRUE

)

Edges_CLPN_t1t2<-Network_CL_adj2$graph

Edges_CLPN_t1t2<-as.data.frame(Edges_CLPN_t1t2)

write_xlsx(

x = Edges_CLPN_t1t2,

path = "Edges_CLPN_t1t2.xlsx",

col_names = TRUE

)

#2.7 Draw the autoregressive coefficient graph (Figure S4)

nodelist <- c("DEP", "SOM", "ANX", "SQQ", "DIS", "PaS", "AcS", "SuI", "SuT")

NETauto_adj <- diag(Network_CL_adj$graph)

NETauto_adj1 <- diag(Network_CL_adj2$graph)

data <- data.frame(

Symptom = factor(nodelist, levels = nodelist),

Value1 = NETauto_adj,

Value2 = NETauto_adj1

)

plot_data <- data %>%

pivot_longer(

cols = c(Value1, Value2),

names_to = "Network",

values_to = "Coefficient"

) %>%

mutate(

Network = ifelse(Network == "Value1", "T0→T1 Network", "T1→T2 Network")

)

ggplot(plot_data, aes(x = Coefficient, y = Symptom, group = Network)) +

geom_path(

aes(linetype = Network, color = Network),

size = 1.2,

show.legend = TRUE

) +

geom_point(

aes(shape = Network, fill = Network),

size = 4,

color = alpha("black", 0)

) +

scale_linetype_manual(

name = "Network",

values = c("T0→T1 Network" = "solid", "T1→T2 Network" = "solid")

) +

scale_color_manual(

name = "Network",

values = c("T0→T1 Network" = "red", "T1→T2 Network" = "#00C0C0")

) +

scale_shape_manual(

name = "Network",

values = c("T0→T1 Network" = 21, "T1→T2 Network" = 21) # 圆形和三角形

) +

scale_fill_manual(

name = "Network",

values = c("T0→T1 Network" = "red", "T1→T2 Network" = "#00C0C0")

) +

labs(

x = "Autoregressive Edge",

y = "Symptom"

) +

theme_bw() +

theme(

panel.grid.major.x = element_line(color = "grey90", size = 0.5),

panel.grid.minor.x = element_blank(),

panel.grid.major.y = element_line(color = "grey90", linetype = "dotted", size = 0.5),

plot.title = element_text(size = 16, face = "bold", hjust = 0.5),

axis.title = element_text(size = 14, face = "bold"),

axis.text = element_text(size = 12),

legend.position = "right",

legend.title = element_text(face = "bold", size = 12),

legend.text = element_text(size = 11),

legend.key.height = unit(0.8, "cm"),

legend.key = element_rect(color = NA, fill = NA),

legend.background = element_blank(),

legend.box.just = "center"

) +

geom_vline(

xintercept = 0,

linetype = "dashed",

color = "grey40",

size = 0.7

) +

scale_x_continuous(

expand = expansion(mult = c(0.1, 0.1)),

breaks = scales::pretty_breaks(n = 8)

)

## multiple imputation for sensitivity analysis.

# 1. Ensure the original data is loaded and backed up

df_original <- data_with_missingsav # Your original dataframe

df_clean <- df_original # Create a working copy

# Get the current column name vector

old_names <- colnames(df_clean)

new_names <- old_names # Initialize with original column names

# T0 Baseline symptoms (Columns 2-10)

new_names[old_names == "BDI13_index_total"] <- "DEP_T0"

new_names[old_names == "BAI_F1_index"] <- "SOM_T0"

new_names[old_names == "BAI_F2_index"] <- "ANX_T0"

new_names[old_names == "AIS_F1_INDEX"] <- "SQQ_T0"

new_names[old_names == "AIS_F2_INDEX"] <- "DIS_T0"

new_names[old_names == "ESS_F1_INDEX"] <- "Pas_T0"

new_names[old_names == "ESS_F2_INDEX"] <- "AcS_T0"

new_names[old_names == "BSI_F1_INDEX"] <- "SuI_T0"

new_names[old_names == "BSI_F2_INDEX"] <- "SuT_T0"

# T1 symptoms (Columns 16-24)

new_names[old_names == "BDI13_1st_all"] <- "DEP_T1"

new_names[old_names == "BAI_F1_1st"] <- "SOM_T1"

new_names[old_names == "BAI_F2_1st"] <- "ANX_T1"

new_names[old_names == "AIS_F1_1st"] <- "SQQ_T1"

new_names[old_names == "AIS_F2_1st"] <- "DIS_T1"

new_names[old_names == "ESS_F1_1st"] <- "Pas_T1"

new_names[old_names == "ESS_F2_1st"] <- "AcS_T1"

new_names[old_names == "BSI_F1_1st"] <- "SuI_T1"

new_names[old_names == "BSI_F2_1st"] <- "SuT_T1"

# T2 symptoms (Columns 25-33)

new_names[old_names == "BDI13_3rd_all"] <- "DEP_T2"

new_names[old_names == "BAI_F1_3rd"] <- "SOM_T2"

new_names[old_names == "BAI_F2_3rd"] <- "ANX_T2"

new_names[old_names == "AIS_F1_3rd"] <- "SQQ_T2"

new_names[old_names == "AIS_F2_3rd"] <- "DIS_T2"

new_names[old_names == "ESS_F1_3rd"] <- "Pas_T2"

new_names[old_names == "ESS_F2_3rd"] <- "AcS_T2"

new_names[old_names == "BSI_F1_3rd"] <- "SuI_T2"

new_names[old_names == "BSI_F2_3rd"] <- "SuT_T2"

# Apply new column names

colnames(df_clean) <- new_names

library(mice)

# Explicitly specify variables to be imputed (all symptom variables at T1 and T2)

vars_to_impute <- c(t1_vars, t2_vars)

# Configure imputation methods

# Initialize: Set imputation method for all variables to empty string "", meaning no imputation by default

method_vector <- rep("", ncol(df_clean))

names(method_vector) <- colnames(df_clean)

# Set only target variables (T1, T2 symptoms) to "pmm" method for imputation

# Patient ID, baseline variables, etc. will remain as "" and not be imputed, but can be used as predictors

method_vector[vars_to_impute] <- "pmm"

# Perform imputation

imputed_final <- mice(df_clean,

method = method_vector,

m = 20,

maxit = 10,

seed = 2024,

printFlag = TRUE)

# Check convergence

plot(imputed_final)

# Check distribution of imputed values

densityplot(imputed_final, ~ DEP_T1 + DEP_T2) # Check key variables

library(bootnet)

nodelist <- c("DEP", "SOM", "ANX", "SQQ", "DIS", "Pas", "AcS", "SuI", "SuT")

fit_network_simple <- function(data) {

# Select data using standardized variable names, absolutely reliable

T1_data <- data[, paste0(nodelist, "_T1")]

T2_data <- data[, paste0(nodelist, "_T2")]

# Set node labels

colnames(T1_data) <- nodelist

colnames(T2_data) <- nodelist

# Fit networks

T1_net <- estimateNetwork(T1_data, default = "EBICglasso", labels = nodelist)

T2_net <- estimateNetwork(T2_data, default = "EBICglasso", labels = nodelist)

return(list(T1 = T1_net, T2 = T2_net))

}

# Test the function

test_data <- complete(imputed_final, 1)

test_result <- fit_network_simple(test_data)

cat("✓ Network analysis function test successful!\n")

# Analyze all 20 imputed datasets

all_results <- with(imputed_final, fit_network_simple(data))

# Extract and compare core metrics (e.g., T2 network centrality rankings)

# You need to write specific comparison logic here to compare with your original analysis results

# Check actual column names in the imputed dataframe

test_data <- complete(imputed_final, 1)

cat("Column names in the imputed dataframe:\n")

print(colnames(test_data))

# Check if our expected T1 column names actually exist

expected_t1_cols <- paste0(nodelist, "_T1")

expected_t2_cols <- paste0(nodelist, "_T2")

cat("Expected T1 column names:\n")

print(expected_t1_cols)

cat("Do these column names exist in the data:\n")

print(expected_t1_cols %in% colnames(test_data))

cat("Expected T2 column names:\n")

print(expected_t2_cols)

cat("Do these column names exist in the data:\n")

print(expected_t2_cols %in% colnames(test_data))

# Embed nodelist directly inside the function to avoid environment variable dependency

fit_network_fixed <- function(data) {

# Define nodelist inside the function to ensure self-containment

nodelist <- c("DEP", "SOM", "ANX", "SQQ", "DIS", "Pas", "AcS", "SuI", "SuT")

# Construct complete variable names

t1_vars <- paste0(nodelist, "_T1")

t2_vars <- paste0(nodelist, "_T2")

# Validate variable existence

if(!all(t1_vars %in% colnames(data)) | !all(t2_vars %in% colnames(data))) {

stop("Required variable columns not found in the data")

}

# Extract data

T1_symptoms <- data[, t1_vars, drop = FALSE]

T2_symptoms <- data[, t2_vars, drop = FALSE]

# Set column names

colnames(T1_symptoms) <- nodelist

colnames(T2_symptoms) <- nodelist

# Fit networks

T1NET <- estimateNetwork(T1_symptoms, default = "EBICglasso",

threshold = TRUE, labels = nodelist)

T2NET <- estimateNetwork(T2_symptoms, default = "EBICglasso",

threshold = TRUE, labels = nodelist)

return(list(T1_Network = T1NET, T2_Network = T2NET))

}

# Test with a single dataset

test_data <- complete(imputed_final, 1)

test_result <- fit_network_fixed(test_data)

cat("✓ Single dataset test successful!\n")

cat("T1 network nodes:", paste(colnames(test_result$T1_Network$graph), collapse = ", "), "\n")

# This should now work properly

all_results <- with(imputed_final, fit_network_fixed(data))

cat("✓ Multiple imputation network analysis completed!\n")
