# Supplementary material for: Network dynamics of depression, anxiety, sleep disturbances, and suicidal symptoms in Chinese adolescents: a longitudinal cross-sectional and cross-lagged panel network analysis
Source: Psychol Med. 2026 Jan 23;56:e30. doi: 10.1017/S0033291726103183 (PMC12885338; doi:10.1017/S0033291726103183)
Supplement: Sun et al. supplementary material 2 — Sun et al. supplementary material [file S0033291726103183sup002.docx]

**Supplementary materials**

***Table S1*** Symptom dimension assessment index

***Table S2.*** Comparison of Baseline Characteristics Between Participants Retained and Those Lost to Follow-up at Each Time Point

***Figure S1*.** Accuracy of edges weights of the cross-sectional networks from T0 to T2. Bootstrapped 95% confidence intervals around each edge weight. Red lines indicate the edge weight in the estimated sample network.

***Figure S2***. Bootstrapped difference tests for centrality indices related to cross-sectional networks of T0 (first row), T1(middle row) and T2 (last row).

***Figure S3*.** Correlation stability of centrality indices related to cross-sectional networks from T0 to T2.

***Table S3.*** Comparisons of Network Structure at 3 time points.

***Table S4.*** Cross-lagged LASSO regression predictive effects between T0 and T1

***Figure S4*.** Autoregressive edges for each symptom in the T0→T1 and T1→T2 network.

***Figure S5.*** Accuracy of edges weights of the cross-lagged networks in the T0→T1 and T1→T2 network. Red dots and lines are edge weights from the samples. Black dots and lines are edge weights that are generated based on 1000 random bootstrap samples.

***Figure S6.*** Bootstrapped difference tests between edge weights in the T0→T1 and T1→T2 network. Non-significant and significant (*p*<0.05) differences between edges are indicated by grey and black boxes,

***Figure S7.*** Bootstrapped difference tests for centrality indices related to the cross-lagged networks of T0→T1 (upper row) and T1→T2 (lower row).

***Figure S8.*** Stability of centrality measures in T0→T1 and T1→T2 networks.

***Table S5.*** Comparative Ranking Stability of Network Centrality Metrics: Original versus Pooled Data across T1 and T2 Assessments

***Figure S9.*** Comparative Stability of Symptom Node Rankings in Cross-sectional Networks: Original versus Pooled Data across Multiple Imputations

***Figure S10.*** Sensitivity Analysis of Multiple Imputation: Edge Weight Consistency between Original and Pooled Data in Cross-sectional Networks

***Table S6.*** Comparative Ranking Stability of Network Centrality Metrics: Original versus Pooled Data across T1 and T2 Assessments

***Table S7.*** Comparative Ranking Stability of Cross-Lagged Panel Network Centrality Metrics: Original versus Pooled Data across T0→T1 and T1→T2 Networks

***Figure S11.*** Comparative Stability of Symptom Node Rankings in Cross-Lagged Panel Network : Original versus Pooled Data across Multiple Imputations

***Figure S12.*** Sensitivity Analysis of Multiple Imputation: Cross-lagged Paths Consistency between Original and Pooled Data

***Table S1* Symptom dimension assessment index**

| **Symptoms** | **Labels** | **Tools** | **Dimension** |
| --- | --- | --- | --- |
| Depression symptom | DEP | BDI-13 | Depression |
| Somatic symptom | SOM | BAI-21 | Anxiety |
| Subjective anxiety | ANX | BAI-21 | Anxiety |
| Sleep quantity and quality | SQQ | AIS-9 | Insomnia |
| Daytime insomnia symptoms | DIS | AIS-9 | Insomnia |
| Passive sleepiness | PaS | ESS-9 | Sleepiness |
| Active sleepiness | AcS | ESS-9 | Sleepiness |
| Suicide ideation | SuI | BSI-19 | Suicide |
| Suicide tendency | SuT | BSI-19 | Suicide |

Abbreviation: BDI― Beck Depression Inventory, BAI― Beck Anxiety Inventory, AIS― Athens Insomnia Scale, ESS― Epworth Sleepiness Scale, BSI―Beck Scale for Suicide Ideation

***Table S2.*** Comparison of Baseline Characteristics Between Participants Retained and Those Lost to Follow-up at Each Time Point

| **Variables** | **T0** | | | |  | **T1** | | | |  | **T2** | | | |
| --- | --- | --- | --- | --- | --- | --- | --- | --- | --- | --- | --- | --- | --- | --- |
|  | **Retained (N=649)** | **Lost (N=148)** | **Statistics** | **P** |  | **Retained (N=458)** | **Lost (N=339)** | **Statistics** | **P** |  | **Retained (N=277)** | **Lost (N=520)** | **Statistics** | **P** |
| Age | 18.4(3.3) | 18.7(3) | t=1.074 | 0.283 |  | 18.7(3.1) | 18.1(3.3) | t=-2.883 | 0.004* |  | 18.8(3.1) | 18.2(3.3) | t=-2.465 | 0.012* |
| Gender |  |  | χ2=0.557 | 0.456 |  |  |  | χ2=0.174 | 0.676 |  |  |  | χ2=0.207 | 0.649 |
| Male | 150(23.1%) | 30(20.3%) |  |  |  | 101(22.1%) | 79(23.3%) |  |  |  | 60(21.7%) | 120(23.1%) |  |  |
| Female | 499(76.9%) | 118(79.7%) |  |  |  | 357(77.9%) | 260(76.7%) |  |  |  | 217(78.4%) | 400(76.9%) |  |  |
| Education |  |  | χ2=2.656 | 0.266 |  |  |  | χ2=13.959 | 0.001* |  |  |  | χ2=13.372 | 0.001* |
| Junior High or Lower | 154(23.7%) | 28(19.2%) |  |  |  | 97(21.3%) | 85(25.1%) |  |  |  | 51(18.5%) | 131(25.2%) |  |  |
| High School | 246(37.9%) | 52(35.6%) |  |  |  | 153(33.6%) | 145(42.8%) |  |  |  | 92(33.3%) | 206(39.7%) |  |  |
| University or Above | 249(38.4%) | 66(45.2%) |  |  |  | 206(45.2%) | 109(32.2%) |  |  |  | 133(48.2%) | 182(35.1%) |  |  |
| Duration | 2.5(2.2) | 1.9(2) | t=2.956 | 0.003* |  | 2.4(2.2) | 2.3(2.2) | t=0.361 | 0.718 |  | 2.3(2.1) | 2.4(2.2) | t=0.174 | 0.862 |
| Antidepressants​ | 422(65%) | 89(60.1%) | χ2=1.252 | 0.263 |  | 303(66.2%) | 208(61.4%) | χ2=1.951 | 0.162 |  | 191(69%) | 320(61.5%) | χ2=4.318 | 0.038* |
| Benzodiazepines​ | 282(43.5%) | 58(39.2%) | χ2=4.318 | 0.038* |  | 199(43.4%) | 141(41.6%) | χ2=0.275 | 0.6 |  | 117(42.2%) | 223(42.9%) | χ2=0.031 | 0.861 |
| Mood Stabilizers​ | 215(33.1%) | 51(34.5%) | χ2=0.096 | 0.757 |  | 169(36.9%) | 97(28.6%) | χ2=6.015 | 0.014* |  | 107(38.6%) | 159(30.6%) | χ2=5.269 | 0.022* |
| Antipsychotics | 331(51%) | 72(48.6%) | χ2=0.267 | 0.605 |  | 248(54.1%) | 155(45.7%) | χ2=5.533 | 0.019* |  | 153(55.2%) | 250(48.1%) | χ2=3.704 | 0.054 |

* P<0.05


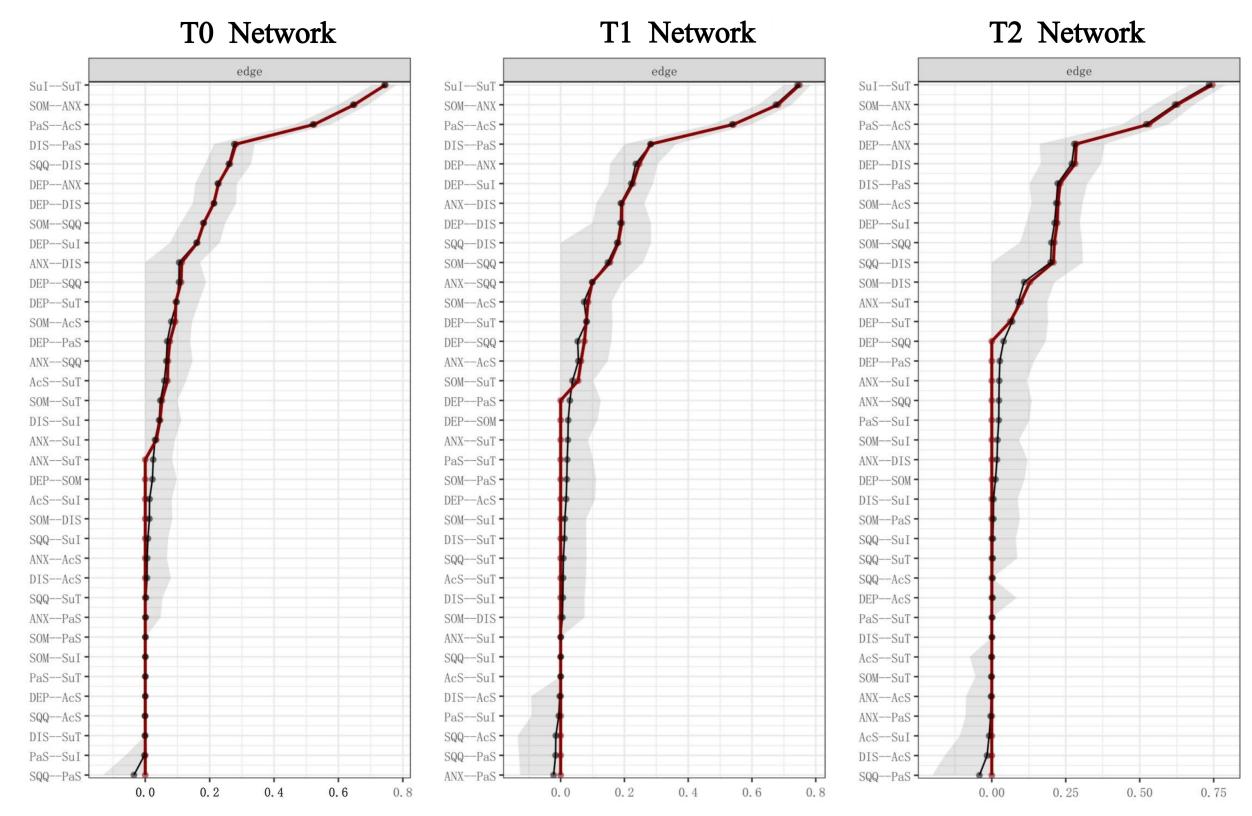


***Figure S1*.** Accuracy of edges weights of the cross-sectional networks from T0 to T2. Bootstrapped 95% confidence intervals around each edge weight. Red lines indicate the edge weight in the estimated sample network. Node abbreviations: DEP―Depression symptoms, SOM―Somatic symptom, ANX―Subjective anxiety, SQQ―Sleep quantity and quality, DIS―Daytime insomnia symptoms, Pas―Passive Sleepiness, AcS―Active Sleepiness, SuI―Suicide Ideation, SuT―Suicide tendency.


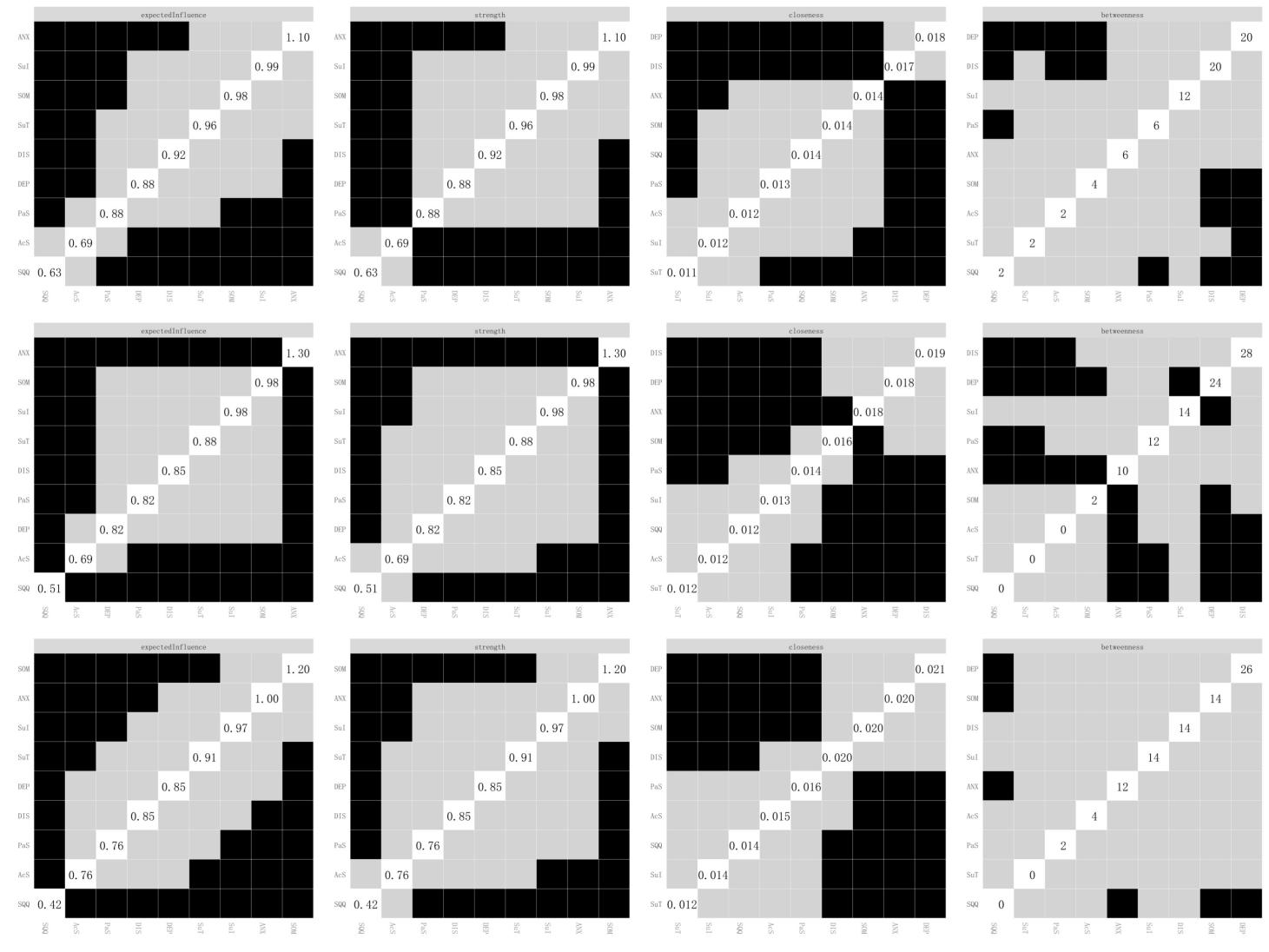


***Figure S2***. Bootstrapped difference tests for centrality indices related to cross-sectional networks of T0 (first row), T1(middle row) and T2 (last row). Grey boxes indicate non-significant differences (*p*>.050). Values in the diagonal are the raw centrality estimates for the given node. Node abbreviations: DEP―Depression symptoms, SOM―Somatic symptom, ANX―Subjective anxiety, SQQ―Sleep quantity and quality, DIS―Daytime insomnia symptoms, Pas―Passive Sleepiness, AcS―Active Sleepiness, SuI―Suicide Ideation, SuT―Suicide tendency.


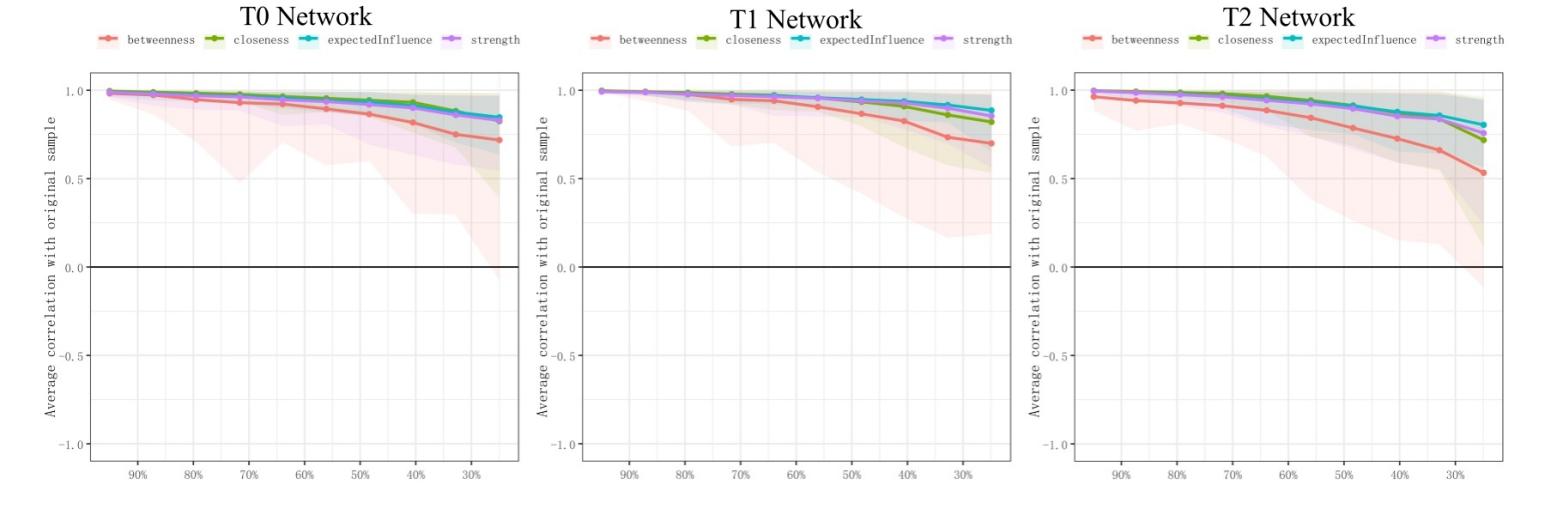


***Figure S3*.** Correlation stability of centrality indices related to cross-sectional networks from T0 to T2.

***Table S2.*** Comparisons of Network Structure at 3 time points.

| **Network 1** | **Network 2** | **Global Strength Invariance Test** | | | |  | **Network Invariance Test** | |
| --- | --- | --- | --- | --- | --- | --- | --- | --- |
|  |  | **Global Strength of Network 1** | **Global Strength of Network 2** | **S** | **P-value** |  | **M** | **P-value** |
| T0 Network | T1 Network | 4.009 | 3.906 | 0.102 | 1.000 |  | 0.082 | 1.000 |
| T0 Network | T2 network | 4.009 | 3.857 | 0.152 | 1.000 |  | 0.130 | 1.000 |
| T1 Network | T2 network | 3.906 | 3.857 | 0.049 | 1.000 |  | 0.192 | 0.417 |

Note.P-values were adjusted using the Bonferroni correction.

***Table S3.*** Cross-lagged LASSO regression predictive effects between T0 and T1

|  | | **Outcome variables: symptoms at T1** | | | | | | | | |
| --- | --- | --- | --- | --- | --- | --- | --- | --- | --- | --- |
|  |  | **DEP** | **SOM** | **ANX** | **SQQ** | **DIS** | **PaS** | **AcS** | **SuI** | **SuT** |
| **Predictor variables: symptoms at T0** | **DEP** | 0.426 | 0.000 | 0.000 | 0.000 | 0.000 | 0.011 | 0.000 | 0.000 | 0.015 |
|  | **SOM** | 0.019 | 0.586 | 0.155 | 0.059 | 0.000 | 0.000 | 0.023 | 0.017 | 0.027 |
|  | **ANX** | 0.230 | 0.083 | 0.472 | 0.000 | 0.078 | 0.019 | 0.006 | 0.000 | 0.000 |
|  | **SQQ** | 0.123 | 0.044 | 0.007 | 0.357 | 0.032 | 0.037 | 0.006 | 0.000 | 0.048 |
|  | **DIS** | 0.431 | 0.000 | 0.231 | 0.060 | 0.271 | 0.066 | 0.000 | 0.022 | 0.000 |
|  | **PaS** | 0.004 | 0.000 | 0.000 | 0.007 | 0.079 | 0.537 | 0.041 | 0.005 | 0.092 |
|  | **AcS** | 0.647 | 0.683 | 0.509 | 0.004 | 0.011 | 0.208 | 0.470 | 0.140 | 0.120 |
|  | **SuI** | 0.263 | 0.127 | 0.082 | 0.028 | 0.068 | 0.036 | 0.000 | 0.520 | 0.590 |
|  | **SuT** | 0.114 | 0.000 | 0.000 | 0.017 | 0.000 | 0.040 | 0.009 | 0.076 | 0.478 |

Note. Values between the symptoms are unstandardized regression coefficients (B) based on Linear regression models. Node abbreviations: DEP―Depression symptoms, SOM―Somatic symptom, ANX―Subjective anxiety, SQQ―Sleep quantity and quality, DIS―Daytime insomnia symptoms, Pas―Passive Sleepiness, AcS―Active Sleepiness, SuI―Suicide Ideation, SuT―Suicide tendency.

***Table S4.*** Cross-lagged LASSO regression predictive effects between T1 and T2

|  | | **Outcome variables: symptoms at T2** | | | | | | | | |
| --- | --- | --- | --- | --- | --- | --- | --- | --- | --- | --- |
|  |  | **DEP** | **SOM** | **ANX** | **SQQ** | **DIS** | **PaS** | **AcS** | **SuI** | **SuT** |
| **Predictor variables: symptoms at T1** | **DEP** | 0.297 | 0.000 | 0.000 | 0.000 | 0.000 | 0.000 | 0.000 | 0.000 | 0.000 |
|  | **SOM** | 0.000 | 0.515 | 0.064 | 0.000 | 0.001 | 0.000 | 0.011 | 0.000 | 0.000 |
|  | **ANX** | 0.210 | 0.024 | 0.496 | 0.014 | 0.070 | 0.000 | 0.000 | 0.000 | 0.000 |
|  | **SQQ** | 0.000 | 0.000 | 0.000 | 0.361 | 0.032 | 0.000 | 0.000 | 0.000 | 0.000 |
|  | **DIS** | 0.000 | 0.000 | 0.000 | 0.000 | 0.136 | 0.000 | 0.000 | 0.000 | 0.000 |
|  | **PaS** | 0.000 | 0.000 | 0.000 | 0.000 | 0.000 | 0.351 | 0.000 | 0.000 | 0.000 |
|  | **AcS** | 0.000 | 0.000 | 0.000 | 0.000 | 0.000 | 0.000 | 0.256 | 0.000 | 0.000 |
|  | **SuI** | 0.000 | 0.000 | 0.000 | 0.000 | 0.000 | 0.000 | 0.000 | 0.102 | 0.000 |
|  | **SuT** | 0.065 | 0.000 | 0.036 | 0.000 | 0.000 | 0.000 | 0.000 | 0.155 | 0.498 |

Note. Values between the symptoms are unstandardized regression coefficients (B) based on Linear regression models. Node abbreviations: DEP―Depression symptoms, SOM―Somatic symptom, ANX―Subjective anxiety, SQQ―Sleep quantity and quality, DIS―Daytime insomnia symptoms, Pas―Passive Sleepiness, AcS―Active Sleepiness, SuI―Suicide Ideation, SuT―Suicide tendency.


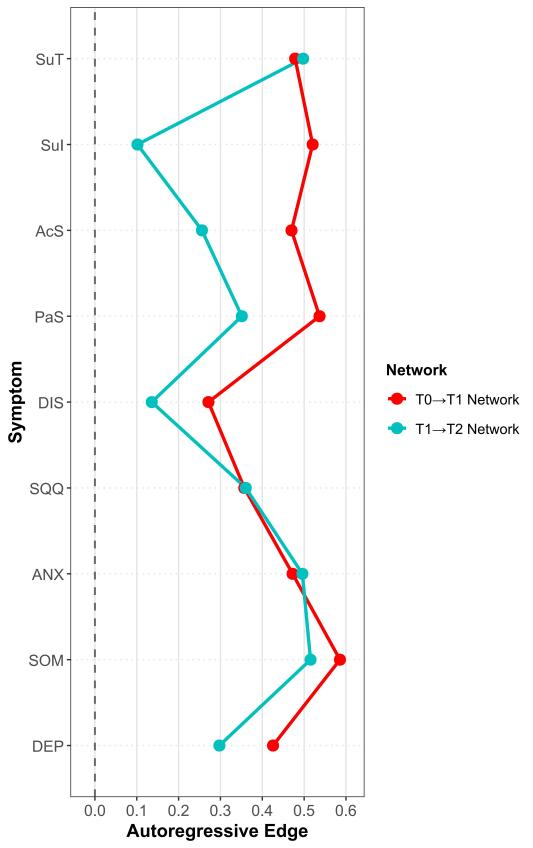


***Figure S4*.** Autoregressive edges for each symptom in the T0→T1 and T1→T2 network. Node abbreviations: DEP―Depression symptoms, SOM―Somatic symptom, ANX―Subjective anxiety, SQQ―Sleep quantity and quality, DIS―Daytime insomnia symptoms, Pas―Passive Sleepiness, AcS―Active Sleepiness, SuI―Suicide Ideation, SuT―Suicide tendency.


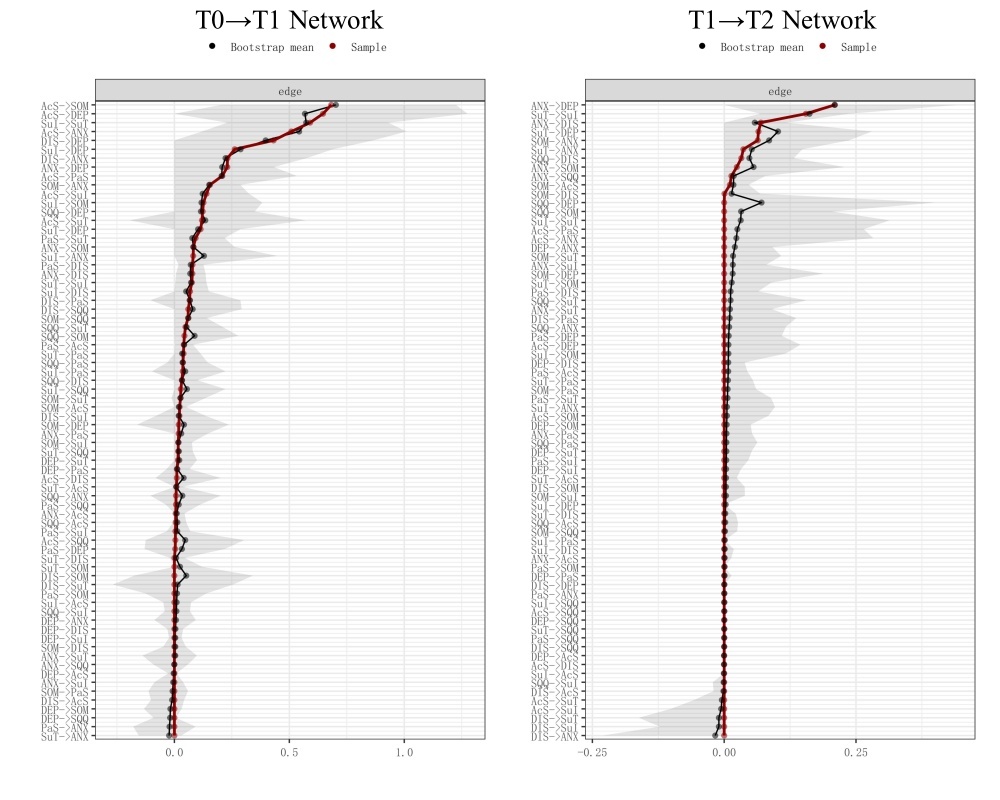


***Figure S5.*** Accuracy of edges weights of the cross-lagged networks in the T0→T1 and T1→T2 network. Red dots and lines are edge weights from the samples. Black dots and lines are edge weights that are generated based on 1000 random bootstrap samples. Node abbreviations: DEP―Depression symptoms, SOM―Somatic symptom, ANX―Subjective anxiety, SQQ―Sleep quantity and quality, DIS―Daytime insomnia symptoms, Pas―Passive Sleepiness, AcS―Active Sleepiness, SuI―Suicide Ideation, SuT―Suicide tendency.


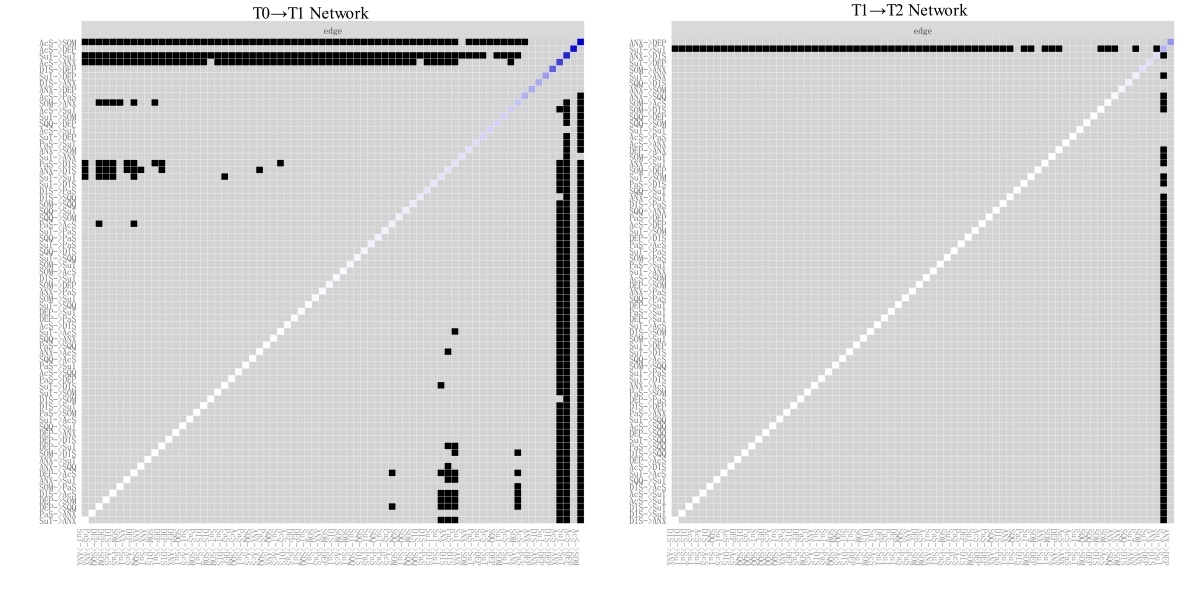


***Figure S6.*** Bootstrapped difference tests between edge weights in the T0→T1 and T1→T2 network. Non-significant and significant (*p*<0.05) differences between edges are indicated by grey and black boxes, respectively. Node abbreviations: DEP―Depression symptoms, SOM―Somatic symptom, ANX―Subjective anxiety, SQQ―Sleep quantity and quality, DIS―Daytime insomnia symptoms, Pas―Passive Sleepiness, AcS―Active Sleepiness, SuI―Suicide Ideation, SuT―Suicide tendency.


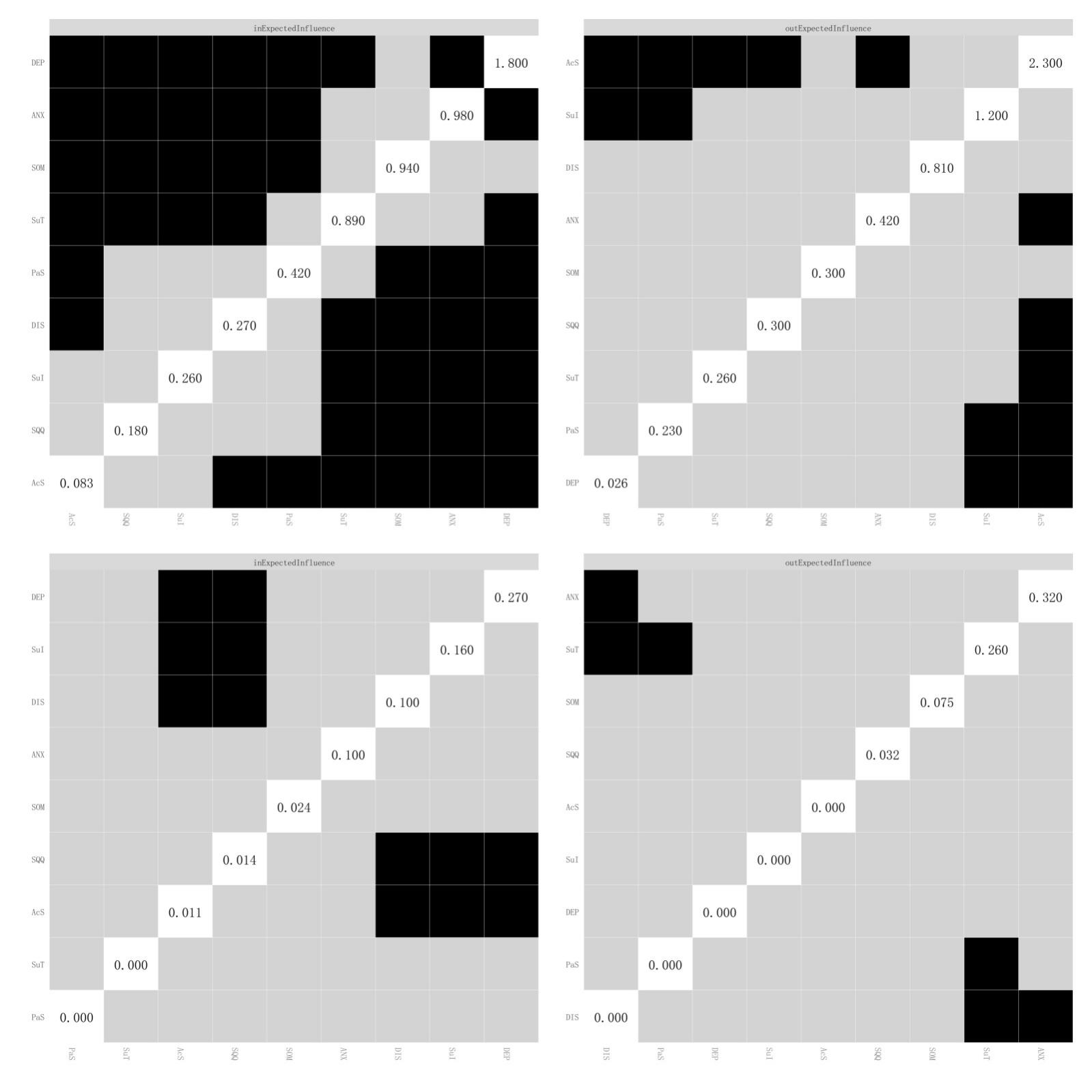


***Figure S7.*** Bootstrapped difference tests for centrality indices related to the cross-lagged networks of T0→T1 (upper row) and T1→T2 (lower row). Non-significant and significant (*p*<0.05) differences between edges are indicated by grey and black boxes, respectively. Values in the diagonal are the raw centrality estimates for the given node. Node abbreviations: DEP―Depression symptoms, SOM―Somatic symptom, ANX―Subjective anxiety, SQQ―Sleep quantity and quality, DIS―Daytime insomnia symptoms, Pas―Passive Sleepiness, AcS―Active Sleepiness, SuI―Suicide Ideation, SuT―Suicide tendency.


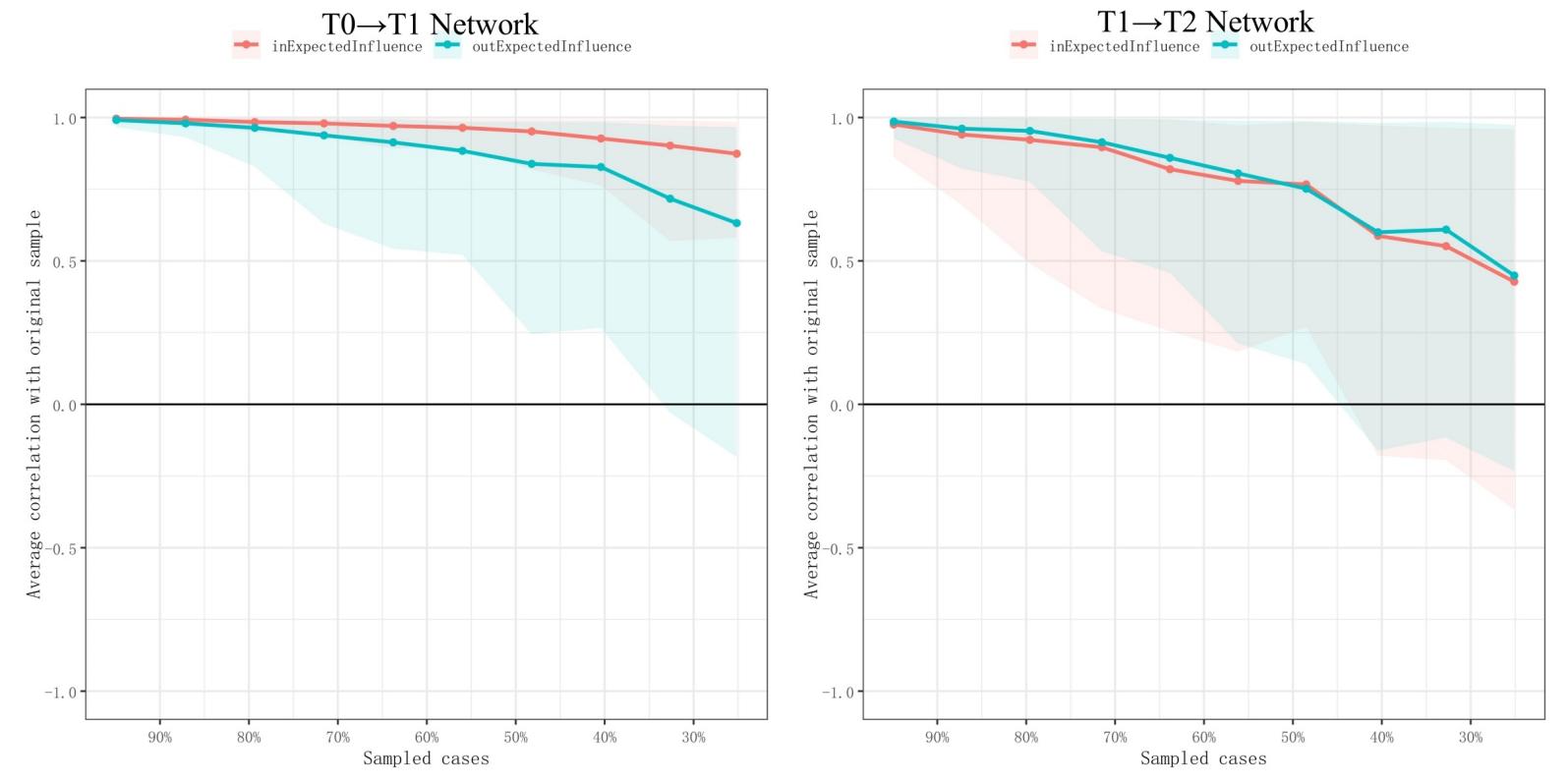


***Figure S8.*** Stability of centrality measures in T0→T1 and T1→T2 networks.

***Table S5.*** Comparative Ranking Stability of Network Centrality Metrics: Original versus Pooled Data across T1 and T2 Assessments

| Time | Node | Strength | | Closeness | | Betweenness | | ExpectedInfluence | |
| --- | --- | --- | --- | --- | --- | --- | --- | --- | --- |
|  |  | Original | Pooled | Original | Pooled | Original | Pooled | Original | Pooled |
| T1 | DEP | 7 | 6 | 2 | 2 | 2 | 1.5 | 7 | 6 |
|  | SOM | 2 | 2 | 4 | 4 | 6 | 5 | 2 | 2 |
|  | ANX | 1 | 1 | 3 | 3 | 5 | 4 | 1 | 1 |
|  | SQQ | 9 | 9 | 7 | 8 | 8 | 8 | 9 | 9 |
|  | DIS | 5 | 7 | 1 | 1 | 1 | 1.5 | 5 | 7 |
|  | PaS | 6 | 5 | 5 | 5 | 4 | 3 | 6 | 5 |
|  | AcS | 8 | 8 | 8 | 6 | 8 | 6 | 8 | 8 |
|  | SuI | 3 | 4 | 6 | 7 | 3 | 8 | 3 | 4 |
|  | SuT | 4 | 3 | 9 | 9 | 8 | 8 | 4 | 3 |
| T2 | DEP | 5 | 5 | 1 | 2 | 1 | 1 | 5 | 5 |
|  | SOM | 1 | 1 | 3 | 3 | 3 | 2 | 1 | 1 |
|  | ANX | 2 | 3 | 2 | 1 | 5 | 3.5 | 2 | 3 |
|  | SQQ | 9 | 9 | 7 | 7 | 8.5 | 7.5 | 9 | 9 |
|  | DIS | 6 | 7 | 4 | 4 | 3 | 3.5 | 6 | 7 |
|  | PaS | 7 | 6 | 5 | 6 | 7 | 7.5 | 7 | 6 |
|  | AcS | 8 | 8 | 6 | 5 | 6 | 6 | 8 | 8 |
|  | SuI | 3 | 2 | 8 | 8 | 3 | 5 | 3 | 2 |
|  | SuT | 4 | 4 | 9 | 9 | 8.5 | 9 | 4 | 4 |





***Figure S9.*** Comparative Stability of Symptom Node Rankings in Cross-sectional Networks: Original versus Pooled Data across Multiple Imputations





***Figure S10.*** Sensitivity Analysis of Multiple Imputation: Edge Weight Consistency between Original and Pooled Data in Cross-sectional Networks

***Table S6.*** Comparative Ranking Stability of Cross-Lagged Panel Network Centrality Metrics: Original versus Pooled Data across T0→T1 and T1→T2 Networks

| **Node** | **T0→T1** | | | | **T1→T2** | | | |
| --- | --- | --- | --- | --- | --- | --- | --- | --- |
|  | **InExpectedInfluence** | | **OutExpectedInfluence** | | **InExpectedInfluence** | | **OutExpectedInfluence** | |
|  | **Original** | **Pooled** | **Original** | **Pooled** | **Original** | **Pooled** | **Original** | **Pooled** |
| **DEP** | 1 | 1 | 9 | 8 | 1 | 1 | 7 | 8 |
| **SOM** | 3 | 4 | 5 | 5 | 5 | 2 | 3 | 4 |
| **ANX** | 2 | 3 | 4 | 4 | 4 | 3 | 1 | 2 |
| **SQQ** | 8 | 8 | 6 | 9 | 6 | 8 | 4 | 3 |
| **DIS** | 6 | 6 | 3 | 3 | 3 | 5 | 7 | 9 |
| **Pas** | 5 | 5 | 8 | 7 | 8.5 | 4 | 7 | 7 |
| **AcS** | 9 | 9 | 1 | 1 | 7 | 7 | 7 | 5 |
| **SuI** | 7 | 7 | 2 | 2 | 2 | 6 | 7 | 6 |
| **SuT** | 4 | 2 | 7 | 6 | 8.5 | 9 | 2 | 1 |





***Figure S11.*** Comparative Stability of Symptom Node Rankings in Cross-Lagged Panel Network : Original versus Pooled Data across Multiple Imputations





***Figure S12.*** Sensitivity Analysis of Multiple Imputation: Cross-lagged Paths Consistency between Original and Pooled Data
